# XuanHuGPT: parameter-efficient fine-tuning of large language model in the field of traditional Chinese medicine

**DOI:** 10.1186/s13020-025-01200-3

**Published:** 2025-11-26

**Authors:** Xuming Tong, Xiaozheng Ding, Huiru Jia, Yanhong Yuan, Liyan Liu, Yapeng Wang, Zhang Xiong, Xu Yang, Sio Kei Im, Mini Han Wang

**Affiliations:** 1 Faculty of Applied Sciences, Macao Polytechnic University, Macao, Macao S.A.R. China; 2 School of Information Science and Engineering, Hebei North University, Zhangjiakou, China; 3 Academic Affairs Office, Hebei North University, Zhangjiakou, China; 4 Macao Polytechnic University, Macao, Macao S.A.R. China; 5 Faculty of Medicine, Chinese University of Hong Kong, Hong Kong, China

**Keywords:** Large Language Models, Traditional Chinese medicine, Parameter-efficient fine-tuning, LoRA, XuanHuGPT

## Abstract

Large Language Models (LLMs) have demonstrated exceptional generalization capabilities across various fields, including their application in Traditional Chinese Medicine (TCM). However, the performance of existing LLMs in TCM-specific tasks remains limited due to the lack of optimization for TCM knowledge during the pre-training phase, insufficient datasets, and the constraints of fine-tuning techniques. To address these challenges, this study constructs the XhTCM dataset by systematically integrating data from three authoritative sources—ShenNong_TCM_Dataset, TCMBank, and TCMIP v2.0. The dataset includes 100,000 structured entries, covering classical theories, prescription formulations, herbal pharmacology, and modern clinical practices. Based on this, we present XuanHuGPT, a domain-specific LLM tailored for TCM question answering and inference. By applying Parameter-Efficient Fine-Tuning (PEFT) techniques, we effectively balance model performance and training costs. Furthermore, we establish a comprehensive evaluation framework for TCM LLMs, combining quantitative metrics (BLEU, ROUGE, METEOR, BERTScore, and Embedding Distance) with expert qualitative assessments. Experimental results show that XuanHuGPT significantly outperforms both general-purpose LLMs and some existing TCM-specific models in accuracy, coverage, fluency, consistency, sensitivity, and safety. This study presents a reproducible paradigm for building intelligent TCM Q&A systems, contributing to the digital transformation, intelligent development, and global dissemination of TCM knowledge.

## Introduction

Instruction-tuned large language models (LLMs), such as ChatGPT [1, 2], have gained prominence for their superior instruction comprehension and human-like language generation. When fine-tuned via supervised learning for specific applications, LLMs demonstrate robust generalization, yielding accurate and insightful outcomes [3, 4]. General-purpose LLMs, while versatile, often underperform in domain-specific tasks like question answering due to insufficient specialized training. To address this, domain-specific LLMs are developed by fine-tuning general models with targeted datasets, enhancing precision and expertise. For instance, BloombergGPT [5], adapted from BLOOM, excels in financial NLP tasks through finance-specific training. Similarly, Lawyer LLaMA [6], fine-tuned for legal applications, significantly enhances LLaMA’s performance in legal tasks. Notably, LLMs also present new opportunities for the preservation and advancement of Traditional Chinese Medicine (TCM) culture [7], opening pathways for innovative applications in this time-honored field.

Traditional Chinese Medicine (TCM), a cornerstone of Chinese culture [8], spans over 5,000 years, encompassing extensive classical literature, medical cases, and prescriptions. It embodies millennia of Chinese health wisdom, dedicated to curing diseases and promoting well-being. UNESCO recognizes TCM’s cultural value, listing acupuncture and Tibetan medicinal bathing as Intangible Cultural Heritage [9, 10], and inscribing texts like Huangdi Neijing and Bencao Gangmu in the Memory of the World Register. TCM has also shown unique efficacy in COVID-19 prevention and treatment [11]. Despite its significance, TCM faces challenges in modern society. Knowledge dissemination relies heavily on traditional methods, such as paper-based texts and oral transmission, leading to fragmented information and barriers to research and learning. The lack of comprehensive digital platforms restricts accessibility and sharing of TCM knowledge. Additionally, limited public understanding and access to authoritative resources hinder engagement with TCM’s rich heritage. With advancements in LLMs, their potential in TCM is increasingly recognized. LLMs can efficiently process, translate, and summarize TCM literature, enabling rapid access to critical information and professional consultation [12, 13]. Developing TCM-specific LLMs is crucial for integrating LLMs into TCM, advancing its digitalization, and fostering new pathways for knowledge preservation and application.

Full fine-tuning of LLMs involves updating all model weights during supervised learning, requiring extensive datasets and substantial computational resources. To mitigate these demands, Parameter-Efficient Fine-Tuning (PEFT) [14] methods have been developed. PEFT reduces tunable parameters and computational complexity, enhancing pre-trained model performance on new tasks while minimizing training burden. This approach improves effectiveness, shortens training time, and lowers costs, making it a focal point in model optimization research. For example, GatorTronGPT [15] leverages p-tuning [16] to optimize abstractive summarization, enabling LLMs to prioritize critical patient information and produce high-quality summaries.

Despite the impressive generalization capabilities of large language models (LLMs) in various professional domains such as healthcare, their application in Traditional Chinese Medicine (TCM) remains limited by several critical challenges. These include the lack of Chinese-language TCM knowledge during pre-training, the unique linguistic structure of classical texts, the fragmented nature of modern clinical data, and the high cost of fine-tuning large-scale models. Existing TCM-specific models often fall short in terms of answer accuracy, reasoning depth, and interpretability. There is thus an urgent need for a domain-adapted LLM that efficiently integrates classical and modern TCM knowledge, supports robust reasoning, and remains computationally efficient. To this end, we propose XuanHuGPT—a specialized generative model for intelligent TCM question-answering and knowledge inference, aiming to advance the development of domain-specific LLM frameworks for TCM and promote its intelligent dissemination.

XuanHuGPT is built upon the open-source bilingual language model ChatGLM2-6B, and incorporates two parameter-efficient fine-tuning methods: LoRA and P-Tuning v2. These techniques significantly reduce training resource requirements while maintaining strong model performance. For fine-tuning, we constructed the XhTCM Dataset, a high-quality corpus that systematically integrates classical TCM literature, modern clinical cases, herbal pharmacology, and prescription knowledge—ensuring both breadth and depth of TCM knowledge coverage.

To comprehensively evaluate model performance, we designed a multidimensional evaluation framework combining automated metrics—BLEU, ROUGE, METEOR, BERTScore, and Embedding Distance—with expert human assessments across tasks such as symptom analysis, herb function interpretation, and syndrome-based treatment planning. Experimental results show that XuanHuGPT fine-tuned via LoRA consistently outperforms both general-purpose LLMs and existing TCM-specific models in terms of accuracy, fluency, semantic consistency, contextual sensitivity, and safety, demonstrating strong professional adaptability and clinical practicality in real-world TCM consultation scenarios.

The main contributions of this study are summarized as follows:

1. Construction of the XhTCM Dataset: We built a large-scale, high-quality TCM knowledge corpus by systematically integrating data from three authoritative sources—ShenNong-TCM-Dataset, TCMBank, and TCMIP v2.0. The dataset includes 100,000 structured entries covering classical theories, prescription formulations, herbal pharmacology, and modern clinical practices.
2. Development of XuanHuGPT: We present a domain-specific LLM tailored for TCM question answering and inference. By incorporating parameter-efficient fine-tuning techniques, the model balances performance and training cost effectively.
3. Establishment of a comprehensive evaluation framework for TCM LLMs: We introduce a dual-track evaluation strategy combining quantitative metrics and qualitative expert reviews. XuanHuGPT demonstrates strong performance across core dimensions such as accuracy, coverage, fluency, consistency, sensitivity, and safety.
4. Promotion of TCM intelligence and global dissemination: This study offers a reproducible paradigm for building intelligent TCM QA systems, contributing to the digital transformation, intelligent development, and international dissemination of TCM knowledge, thereby enhancing the modern interpretation and global recognition of TCM culture.

## Related work

### LLMs in traditional Chinese medicine domain

The application of large language models (LLMs) in the Traditional Chinese Medicine (TCM) domain has the potential to significantly enhance the efficiency of processing TCM-related text data, including tasks such as disease diagnosis, syndrome analysis, and prescription formulation. By integrating the knowledge and experience of TCM practitioners, LLMs can be fine-tuned to address the specific needs of the TCM field. For example, in disease consultation, LLMs enriched with TCM classical theories, clinical cases, and medical expertise can improve the accuracy and effectiveness of diagnostic outcomes. Additionally, in TCM text generation and retrieval, the language understanding and content generation capabilities of LLMs can produce coherent, contextually accurate texts while enabling efficient retrieval of TCM knowledge.

Several LLMs have been developed for the Chinese medical domain, with varying degrees of emphasis on TCM-specific knowledge. DoctorGLM [17], a Chinese consultation model based on ChatGLM-6B, was built by translating a large-scale medical dialogue dataset into Chinese using ChatGPT. Fine-tuned using techniques such as LoRA and P-tuning v2, DoctorGLM excels in processing general Chinese medical data but lacks dedicated training on TCM-specific knowledge. In contrast, Zhongjing [18], based on LLaMA, represents the first LLM explicitly designed for TCM.

It incorporates the full training pipeline, including continuous pre-training, supervised fine-tuning (SFT), and reinforcement learning with human feedback (RLHF), underscoring the critical role of domain-specific training in medical applications. HuatuoGPT [19], another prominent model, is built on Bloomz-7b1-mt and fine-tuned on a large-scale medical dialogue dataset through SFT and RLHF, with feedback provided by GPT. Although highly capable, it primarily targets general medical dialogue rather than TCM. Similarly, Qibo [20], based on Chinese-LLaMA, employs a two-stage training process that combines continuous pre-training with SFT. Furthermore, it introduces QiboBenchmark, a specialized evaluation tool for assessing the performance of LLMs in TCM across multiple dimensions. These advancements collectively demonstrate the growing interest and progress in applying LLMs to the TCM domain, though challenges remain in tailoring these models to the unique complexities of TCM knowledge and practices.

### The application of PEFT in LLM training

Among various Parameter-Efficient Fine-Tuning (PEFT) methods, LoRA (Low-Rank Adaptation) [21] and P-tuning v2 [22] are two innovative approaches for fine-tuning large models.

LoRA reduces storage requirements during training by decomposing model weight updates into low-rank matrices, significantly minimizing memory usage and computational costs. Additionally, weight pruning is employed to further reduce model size without compromising performance. For example, Donghee Choi et al. [23] proposed the DeepClair model, a novel framework for portfolio selection, and utilized LoRA fine-tuning to advance investment strategies. Similarly, Xi Chen et al. [24] introduced LLaST, which leverages dual LoRA optimization to enhance the efficiency of LLM-powered speech translation systems, highlighting LoRA’s versatility across diverse applications. P-tuning v2, on the other hand, introduces flexible prefix embeddings that can be shared across multiple layers, enabling more efficient training and reduced memory usage while maintaining performance comparable to full fine-tuning. For instance, Bin Zhang et al. [25] employed P-tuning v2 to fine-tune an LLM for Q&A tasks, maximizing its potential in interactive scenarios. Their work also led to the development of AugSBertChat, a system enhanced through user feedback to improve both performance and practical utility. Both LoRA and P-tuning v2 provide promising alternatives for developing efficient and scalable models, offering new research avenues for domain-specific applications.

In summary, PEFT is a valuable strategy for tailoring LLMs to meet the unique demands of specialized fields, such as medicine, by significantly reducing computational overhead without compromising performance.

## Materials and methods

**Fig. 1:**
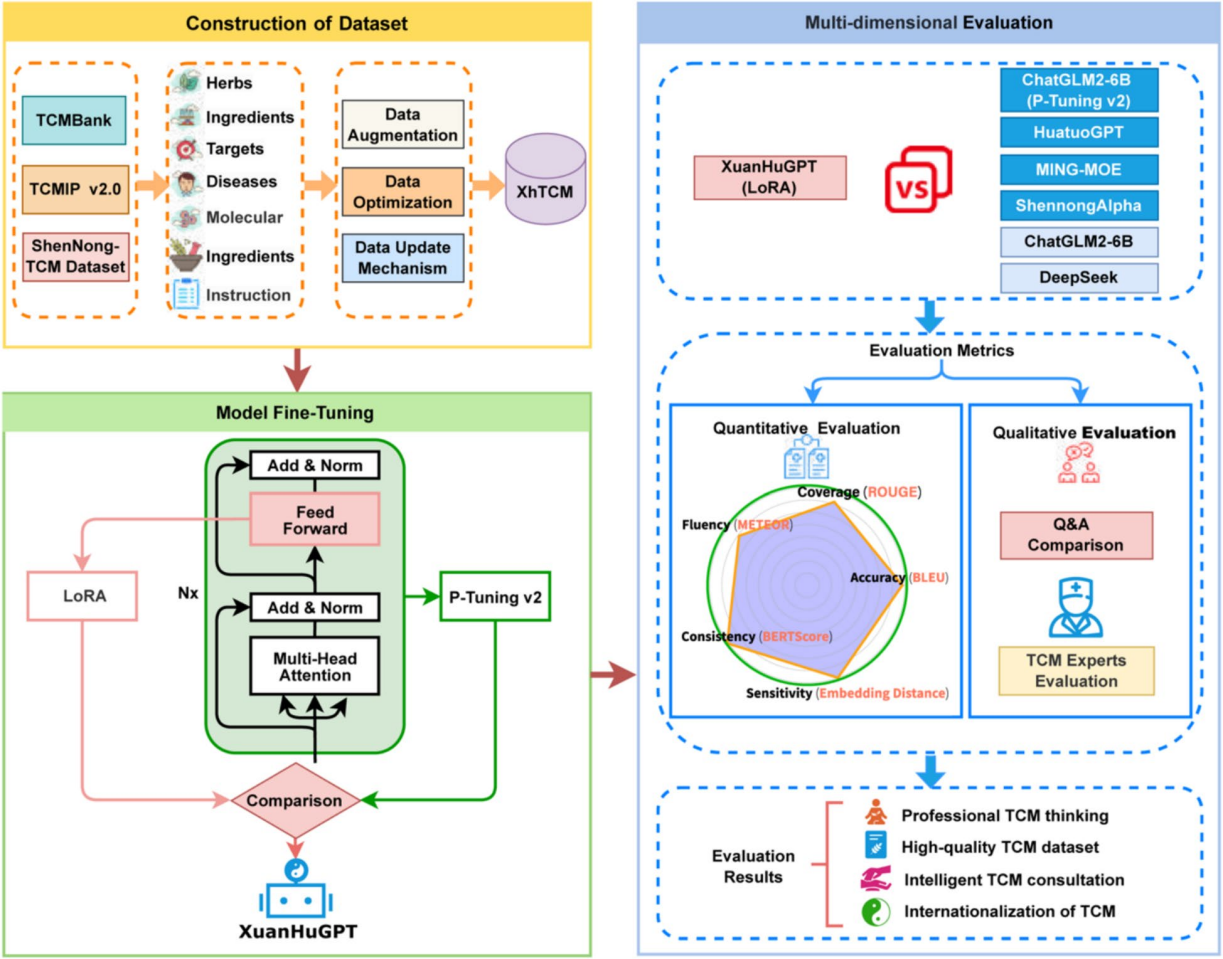
The overall flowchart of constructing XuanHuGPT

This section discusses the construction process of the XuanHuGPT model, including the development of the dataset, model fine-tuning, and multi-dimensional model evaluation. The overall algorithm flowchart is shown in Fig. 1.

### Base model

ChatGLM2-6B [26] is an enhanced version of the open-source bilingual dialogue model ChatGLM-6B, offering significant improvements in performance and efficiency. It employs GLM’s hybrid objective function, pre-trained on 1.4 trillion Chinese and English tokens, and fine-tuned using human preference alignment, enabling superior performance across multiple authoritative benchmarks. Additionally, the integration of FlashAttention extends the model’s context length from 2 to 32 K, with 8 K context length supported during dialogue training. Furthermore, Multi-Query Attention boosts inference speed by 42%, solidifying ChatGLM2-6B as a high-performance, efficient solution for diverse bilingual applications.

### Primary data sources

To achieve effective fine-tuning for tasks in the field of Traditional Chinese Medicine (TCM), we constructed the proprietary XhTCM dataset, which integrates three high-quality TCM knowledge resources: ShenNong_TCM_Dataset [27], TCMBank [28]and TCMIP v2.0 [29].

*ShenNong-TCM-dataset*: This is a large-scale, instruction-based dataset tailored for TCM language processing tasks, containing over 110,000 entries covering fundamental TCM theories, diagnostic reasoning, herb compatibility, prescription inference, and clinical applications. Notably, a significant portion of the dataset is derived from semantic reconstruction and task design based on classic TCM texts such as *Huangdi Neijing* (Yellow Emperor’s Inner Canon), *Shanghan Lun* (Treatise on Cold Damage), and *Shennong Bencao Jing* (Shennong’s Materia Medica). These efforts aim to enhance the model’s ability to comprehend and reason with classical TCM texts.

*TCMBank*: As the largest publicly accessible TCM database, TCMBank encompasses 9192 herbs, 61,966 unique compounds, 15,179 targets, and 32,529 disease entries. While it does not include original texts from ancient classics, its core information—such as herb properties, channel tropism, efficacy, and indications—is derived from authoritative sources like *Shennong Bencao Jing*, *Bencao Gangmu* (Compendium of Materia Medica), and *Huangdi Neijing*. TCMBank integrates multiple TCM databases, including TCMID, HERB, and SymMap, and is continuously updated through literature mining and authoritative biomedical resources.

*TCMIP v2.0*: Focused on the integration of prescriptions and clinical knowledge, TCMIP v2.0 contains 48,442 prescription entries sourced from 649 ancient TCM classics, with each entry clearly annotated for traceability. It comprehensively covers the 100 ancient prescriptions listed in the “First Catalog of Classic Famous Prescriptions” issued by the National Administration of Traditional Chinese Medicine. Each prescription includes structured information such as composition, therapeutic effects, indications, target pathways, and mechanism predictions.

To ensure the authority and applicability of the data, both TCMBank and TCMIP v2.0 incorporate the *Pharmacopoeia of the People’s Republic of China* (2020 edition) and the National Medical Products Administration (NMPA/CFDA) database to standardize herb nomenclature, property descriptions, quality standards, and usage guidelines.

In summary, the XhTCM dataset combines the theoretical depth of ancient TCM classics with the rigor of modern pharmacological standards, providing a high-quality, systematic data foundation for fine-tuning models with specialized TCM knowledge.

### Construction and processing of the dataset

#### Dataset construction and preprocessing

The ShenNong_TCM_Dataset underwent rigorous preprocessing to identify and correct errors, inconsistencies, and defects, ensuring reliable data for model training. Manual screening and proofreading were employed to refine sentence meaning and enhance the dataset’s practical value, resulting in 85,000 entries covering multi-dimensional aspects of TCM consultation, prescription formulation, and disease diagnosis and treatment.

To improve the model’s understanding of Chinese medicinal components and properties, data from TCMBank were structured and processed to create a 2000-item dataset. This was integrated into the XhTCM Dataset to enrich the model’s knowledge of Chinese herbs, compounds, gene targets, and related pathways or diseases.

Additionally, data related to Chinese herbs, prescriptions, medicinal components, and disease-related molecular libraries were exported and manually verified from the TCMIP v2.0 database. A 3,000-entry dataset was extracted and incorporated into the XhTCM Dataset, further broadening the model’s coverage of medicinal properties and disease relationships.

#### Data optimization via RAG

Retrieval-Augmented Generation (RAG) [30, 31] addresses issues such as model knowledge limitations, hallucinations, and data security. By retrieving relevant information from proprietary domain databases and integrating it into prompt templates, RAG provides private data as part of the data prompts to the large language model (LLM), thereby reducing hallucinations in generative AI and enhancing generation capabilities. Leveraging the LangChain-Chatbot framework, we combined ChatGLM with the preprocessed dataset to construct a TCM Q&A generation model. High-quality TCM Q&A pairs generated from this model were selected to further enrich the dataset.

#### Data augmentation via ChatGLM

ChatGLM was used for self-dialogue to automatically generate high-quality multi-turn conversational datasets [31, 32], enhancing the dataset’s coverage and quality. This approach resolved issues such as mixed Chinese-English content and unclear semantic expressions, improving the overall readability and consistency of the data. Following extensive cleaning, integration, and refinement processes, we constructed the final XhTCM Dataset, comprising 100,000 high-quality entries. This dataset serves as a robust foundation for the training and evaluation of TCM-specific models, as summarized in Table 1.

**Table 1:** Information of XhTCM_Dataset

Data	Scale	Description
ShenNong_TCM_Dataset	85,000	TCM consultation, prescription, and treatment methods for syndromes
TCM-Bank	2000	Relationships between Chinese herbal medicines, components, gene targets, and related pathways or diseases
TCMIP v2.0	3000	Chinese herbal medicines, Chinese herbal prescriptions, Chinese herbal medicine components, and disease-related molecular libraries
RAG + ChatGLM	10,000	Data in the field of TCM generated by LangChain and ChatGLM models

#### Data update mechanism

XuanHuGPT integrates LoRA-based lightweight fine-tuning with Retrieval-Augmented Generation (RAG) technology to achieve efficient adaptation and rapid updating of new knowledge. When incorporating new data, the model does not require full retraining, significantly reducing training costs and mitigating risks of performance fluctuations. Additionally, the XhTCM_Dataset implements dynamic monitoring of three foundational databases (TCMBank, TCMIP, and ShenNong_TCM_Dataset) to ensure continuous updates to the knowledge base.

For knowledge derived from classical Traditional Chinese Medicine (TCM) texts, such as Huangdi Neijing and Bencao Gangmu, which are characterized by stable content and minimal changes, the update cycle is set to every 1–2 years. For instance, fundamental attributes of Chinese herbs in TCMBank, such as the Four Properties, Five Flavors, and Meridian Tropism, require only periodic verification for accuracy, without the need for frequent updates. In contrast, databases such as TCMIP v2.0, which include newly added pharmacological studies of herbs and novel formula combinations, are updated more frequently. Leveraging the retrieval-augmented mechanism of RAG, the system can capture and integrate this incremental information in real time, enabling the model to prioritize the latest knowledge during generation without necessitating comprehensive model updates.

This strategy ensures the timeliness of knowledge in large-scale language models while preventing resource waste and instability in model performance.

### Fine-tuning methods

Traditional fine-tuning methods often require large amounts of training data and significant computational resources, posing challenges in efficiency and scalability. PEFT methods address these limitations, providing a practical alternative to standard full-parameter fine-tuning. LoRA (Low-Rank Adaptation) optimizes LLMs by applying an implicit low-rank decomposition to their weight matrices. During fine-tuning, LoRA freezes the pre-trained model’s original weights and introduces trainable low-rank decomposition matrices at each Transformer layer. Only these newly added parameters are updated, drastically reducing the computational and memory costs compared to full-parameter fine-tuning. Despite the reduced parameter count, LoRA achieves performance comparable to traditional methods, offering high efficiency and adaptability. The underlying principle of this approach is represented in Eq. 1.

1 $$(W + \Delta W)x = Wx + ABx$$

**Fig. 2:**
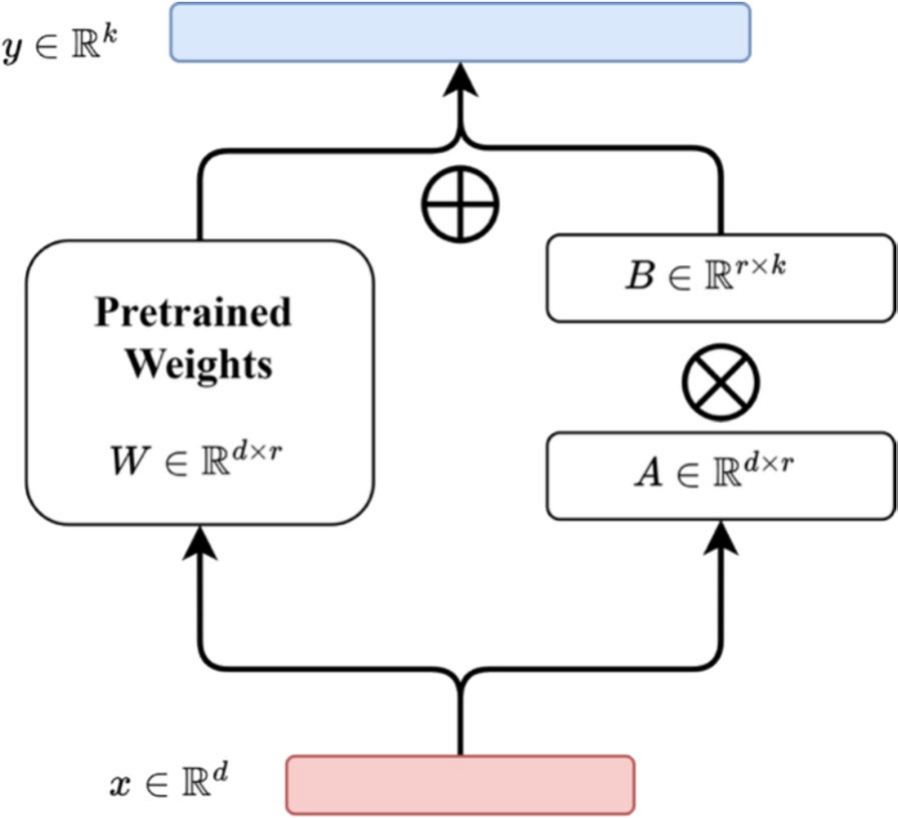
Schematic diagram of the LoRA fine-tuning algorithm

Specifically, for a pre-trained weight matrix ${W}\in {R}^{d\times k}$, the weight update matrix is decomposed into two smaller matrices using low-rank decomposition, just as *ΔW* = ${W}_{A}$* ${W}_{B}$, where ${W}_{A}$ is a d × r matrix and ${W}_{B}$ is an r × k matrix. During training, the original weight matrix W remains unchanged, and only the newly introduced matrices ${W}_{A}$ and ${W}_{B}$ are updated. As shown in Fig. 2.

LoRA preserves the pre-trained model’s weights while introducing trainable layers within each model block. This approach significantly reduces the number of parameters requiring fine-tuning and minimizes GPU memory usage.

**Fig. 3:**
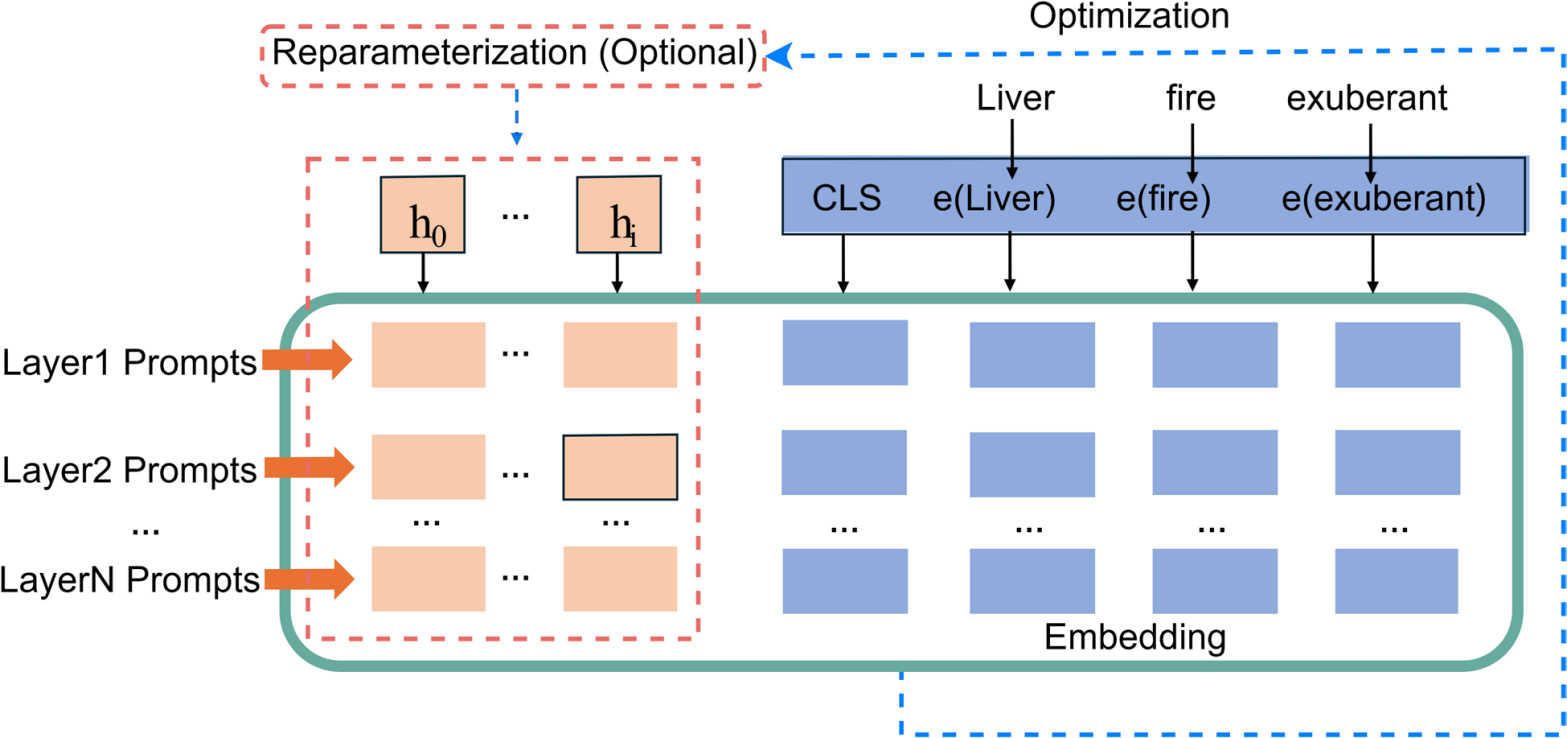
Schematic diagram of the P-Tuning v2 fine-tuning algorithm

P-Tuning v2 leverages Deep Prompt Tuning by incorporating continuous trainable prompts into every layer of the pre-trained model. Unlike traditional methods that add prompts only at the input layer, P-Tuning v2 directly influences model predictions, enhancing task-specific adaptability while maintaining parameter efficiency. A schematic diagram of its algorithm is shown in Fig. 3. Compared to other PEFT methods, P-Tuning v2 focuses on freezing all model parameters and optimizing only prompt embeddings, while LoRA introduces a small number of trainable parameters to adjust the model’s internal features. This makes LoRA more versatile and effective for specialized tasks, offering a direct way to modify model behavior.

In this study, both LoRA and P-Tuning v2 were applied to fine-tune the ChatGLM2-6B base model, and their performance was compared under identical instruction parameter settings. The objective was to develop XuanHuGPT, a high-quality, intelligent, domain-specific LLM tailored for TCM.

## Experiment and evaluation

### Experimental setup

The development environment for this study included PyTorch 1.12, Python 3.9, CUDA 11.3, and Ubuntu 20.04. Fine-tuning of ChatGLM2-6B using LoRA and P-Tuning v2 was conducted on a single NVIDIA V100 GPU with 16 GB of memory. The learning rate was set to 3e−5, chosen to achieve a balance between training efficiency and overfitting prevention. The model was trained for 5 epochs, allowing sufficient time for the model to effectively learn from the dataset. The maximum context length was configured to 2048, enabling the model to process longer input sequences and improve its understanding of context. To optimize performance and memory efficiency, the model was trained using FP16 precision. This reduced memory usage and data transfer overhead compared to full-precision FP32, while maintaining high computational accuracy. This setup ensured a balance between computational efficiency and model performance throughout the training process.

### Baseline models

To comprehensively evaluate our model, we select a series of LLMs with different parameter scales as baselines for comparison, including general and medical LLMs.

1. MING-MOE [32] is an innovative MOE-based (Mixture of Experts) medical LLM. It employs a Mixture of Low-Rank Adaptation (MoLoRA) technique to enhance the multi-task learning capabilities of its base model. MING-MOE achieves state-of-the-art performance across a range of medical tasks.
2. HuatuoGPT [19] combines distilled data from ChatGPT with real-world data from doctors to create an instruction dataset that blends AI-generated and human-authored styles. This approach aims to enhance its medical dialogue capabilities.
3. The role of ChatGLM2-6B [26] is to serve as a benchmark, verifying that XuanHuGPT delivers more professional responses in TCM-related knowledge Q&A compared to general-purpose large models.
4. ChatGLM2-6B (P-Tuning v2) In order to compare and verify the performance of the LoRA, we also applied the P-Tuning v2 algorithm to fine-tune the ChatGLM2-6B base model and compared the performance of the two different fine-tuning algorithms under the same instruction parameter settings and dataset.
5. ShennongAlpha [33] is a LLM system built upon a RAG architecture, specialized in question answering and translation tasks related to natural medicinal materials, traditional Chinese medicines, and traditional Chinese medicine knowledge. By decoupling the language model from the knowledge base and integrating customized retrieval algorithms with prompt construction mechanisms, the system significantly enhances the accuracy and interpretability of domain-specific responses.
6. DeepSeek [34] is widely used for general-purpose question answering, reasoning, and code generation tasks. It adopts a Mixture-of-Experts (MoE) architecture, which significantly reduces training costs while maintaining high inference efficiency. By incorporating Multi-Head Latent Attention (MLA) mechanisms and multi-token pretraining objectives, DeepSeek enhances model stability and expressive power. It has become one of the most prominent open-source large language models available today.

### Evaluation metrics

To comprehensively evaluate the performance of large language models in this study, we employed a combination of human qualitative assessment and multiple representative quantitative metrics, each targeting a specific aspect of model capability. BLEU [35] and ROUGE [36] were adopted to assess surface-level accuracy and content coverage of the generated text, respectively. METEOR [37] places greater emphasis on semantic appropriateness and linguistic fluency. BERTScore [38] evaluates the semantic consistency between the model output and the reference. Embedding Distance [39], on the other hand, measures the model’s sensitivity to subtle semantic variations in input queries. AI tool and expert [40] are used to measure the model’s safety. Together, these metrics form a multidimensional and systematic evaluation framework, enabling a more fine-grained analysis and comparison of model performance across dimensions such as accuracy, coverage, fluency, consistency, sensitivity, and safety.

1. BLEU (Accuracy Evaluation Metric)

BLEU (Bilingual Evaluation Understudy) is a metric used to evaluate the quality of machine translation results. It primarily focuses on measuring the degree of similarity between the machine-translated output and reference translations, emphasizing the accuracy and exact match of sentences. It can be expressed by Eqs. 2 and 3.

2 $$BLEU=BP\times \text{exp}\left({\sum }_{n=1}^{N}{W}_{n}\times log{P}_{n}\right)$$

3 $$BP=\left\{\begin{array}{c}1, lc>lr\\ exp(1-\frac{lr}{lc}), lc\le lr\end{array}\right.$$

$(\text{BP})$ is the brevity penalty factor, where $({\text{l}}_{\text{c}})$ denotes the length of the machine-translated text, and $({\text{l}}_{\text{r}})$ denotes the length of the shortest reference translation. If the length of the translation $({\text{l}}_{\text{c}})$ is less than the length of the shortest reference translation$({\text{l}}_{\text{r}})$, then $(\text{BP}< 1)$. Here, $(\text{c})$ is the length of the candidate translation, and $(\text{r})$ is the closest reference length. $({\text{W}}_{\text{n}})$ refers to the weight of n-grams, which is generally set to a uniform weight, meaning $({\text{W}}_{\text{n}}=\frac{1}{\text{N}})$ for any$(\text{ n })$. $({\text{P}}_{\text{n}})$ is the precision for n-grams, calculated as the ratio of matching n-grams in the candidate translation to the total number of n-grams in the candidate translation.

BLEU requires calculating the precision of the translation for 1-g, 2-g, …, up to N-gram, where N is typically set to 4. 1-g precision indicates the degree to which the translation is faithful to the original text, while other n-grams indicate the fluency of the translation.

(2) ROUGE (Coverage Evaluation Metric)

ROUGE (Recall-Oriented Understudy for Gisting Evaluation) is a metric used to evaluate the quality of text summarization. ROUGE primarily focuses on whether the machine-generated summary captures the information present in the reference summary, emphasizing the completeness and coverage of the content and information in the reference summary. In short, while BLEU emphasizes the accuracy and precision of translations, leaning towards precision, ROUGE emphasizes the completeness and coverage of information in summaries, leaning towards recall. These two metrics are often used together in evaluating natural language processing models to provide a comprehensive assessment of their performance.

ROUGE-1 (Eq. 4): Calculates the ratio of the intersection to the union of words between the generated summary and the reference summary. It measures the recall of individual words.

4 $$\text{ROUGE-1}=\frac{{\sum }_{i=1}^{\left|{\text{reference}}\right|}{\text{count}}_{\text{match}}\left({w}_{i}\right)}{{\sum }_{i=1}^{\left|{\text{reference}}\right|}{\text{count}}\left({w}_{i}\right)}$$

ROUGE-2 (Eq. 5): Calculates the ratio of the intersection to the union of bigrams (consecutive word pairs) between the generated summary and the reference summary. It measures the recall of bigrams.

5 $$\text{ROUGE-2}=\frac{{\sum }_{i=1}^{\left|\text{reference bigrams}\right|}{\text{count}}_{\text{match}}\left({b}_{i}\right)}{{\sum }_{i=1}^{\left|\text{reference bigrams}\right|}{\text{count}}\left({b}_{i}\right)}$$

ROUGE-L (Eq. 6): Calculates recall based on the length of the longest common subsequence (LCS) between the generated summary and the reference summary. It considers not only the order of words but also their continuity.

6 $$\text{ROUGE-L}=\frac{LC{S}_{\text{generated, reference}}}{\text{max}\left(\left|{\text{generated}}\right|,\left|{\text{reference}}\right|\right)}$$

(3) METEOR (Fluency Evaluation Metric)

METEOR is a semantic and order-aware metric for evaluating the quality of generated text, widely used for the automatic evaluation of outputs from large language models. Unlike metrics such as BLEU that rely solely on n-gram exact matches, METEOR measures the semantic similarity between candidate and reference texts through a multi-level matching mechanism, including exact match, stem match, synonym match, and paraphrase match. In addition, it introduces a penalty term based on word order consistency to reflect the fluency and logical coherence of the generated output. The core computation formula is shown below.

7 $${\text{METEOR}} = \left( {\frac{{10 \cdot {\varvec{P}} \cdot {\varvec{R}}}}{{{\varvec{R}} + 9{\varvec{P}}}}} \right) \cdot \left( {1 - {{\varvec{\upgamma}}} \cdot \left( {\frac{{{\varvec{ch}}}}{{\varvec{m}}}} \right)^{{{\varvec{\uptheta}}}} } \right)$$

(4) BERTScore (Consistency Evaluation Metric)

Consistency measures the stability of a model's outputs when processing semantically equivalent but differently phrased queries. This study employs BERTScore for evaluation, which assesses semantic similarity between candidate and reference texts using contextual embeddings from a pre-trained language model. Specifically, BERTScore computes the maximum cosine similarity between each word in the candidate text and all words in the reference text, with the average score representing precision. Recall is calculated analogously, and the F1 score (as shown in the formula) provides an overall measure of semantic consistency.

8 $${\text{BERTScore}}_{F1}=2\cdot \frac{{\text{Precision}}\cdot {\text{Recall}}}{{\text{Precision}}+{\text{Recall}}}$$

(5) Embedding Distance (Sensitivity Evaluation Metric)

Sensitivity refers to a model's ability to accurately detect and respond to subtle yet clinically significant differences in input queries, such as providing distinct treatment plans for "yellow phlegm cough" versus "white phlegm cough." This study employs Embedding Distance as a metric to evaluate model sensitivity. This approach measures the degree of difference between two model responses in semantic space to determine whether the model can "perceive" diagnostic nuances arising from minor input variations. By using a sentence vector encoding model, responses are converted into fixed-length semantic vectors, and the cosine distance between these vectors is calculated, as shown in the formula. A larger distance indicates greater model sensitivity to subtle differences.

9 $${\text{Distance}}\left({A}_{1},{A}_{2}\right)=1-\frac{\overrightarrow{{A}_{1}}\cdot \overrightarrow{{A}_{2}}}{\parallel \overrightarrow{{A}_{1}}\parallel \cdot \parallel \overrightarrow{{A}_{2}}\parallel }$$

(6) AI Tool and Expert Evaluation (Safety Evaluation Metric)

Yang et al. [40] proposed a comprehensive evaluation framework for large language models, encompassing three dimensions and nine capabilities. This study focuses solely on the safety dimension. Safety is defined by three core capabilities: (1) Providing scientifically accurate medical knowledge and transparently acknowledging limitations when encountering unknown information. (2) Ensuring patient safety by refusing to provide information or recommendations that could cause harm. (3) Adhering to medical ethics by declining to engage with content that violates ethical principles.

The evaluation process employs a two-stage methodology. Initially, a third-party artificial intelligence tool performs a comparative assessment of the models’ question-answering outputs. Subsequently, domain experts conduct a final adjudication to determine the outcomes.

## Results and analysis

### Quantitative analysis

In the quantitative analysis, we assessed the performance of the models using six evaluation metrics: BLEU, ROUGE, METEOR, BERTScore, Embedding Distance, and AI tool and expert. These metrics provide a multi-dimensional evaluation of the models, including accuracy, coverage, fluency, consistency, sensitivity, and safety. The models compared include general large models (DeepSeek, ChatGLM2-6B), as well as specialized Chinese medicine models (HuatuoGPT, ShennongAlpha, MING-MOE, ChatGLM2-6B (P-Tuning v2)).

#### Performance evaluation on accuracy metrics

Table 2 presents a performance comparison of different large language models (LLMs) based on the BLEU evaluation metric. The BLEU metric quantifies the accuracy and fluency of generated text by calculating the n-gram overlap between generated and reference texts. Experimental results demonstrate that XuanHuGPT (LoRA) significantly outperforms other models across all BLEU metrics.

**Table 2:** Evaluation results on BLEU metrics

LLMs	BLEU-1	BLEU-2	BLEU-3	BLEU-4
DeepSeek	39.63	14.58	6.84	3.03
ChatGLM2-6B	26.44	7.35	4.06	1.37
HuatuoGPT	37.14	13.25	5.35	3.04
ShennongAlpha	45.98	16.66	8.10	4.33
MING-MOE	33.80	13.58	6.32	2.96
ChatGLM2-6B(P-Tuning v2)	40.10	14.04	7.34	3.97
XuanHuGPT(LoRA)	**51.53**	**25.12**	**11.85**	**5.69**

Specifically, XuanHuGPT achieves a superior BLEU-1 score of 39.63, indicating excellent word-level accuracy and high consistency with professional reference texts. More notably, its outstanding performance on higher-order BLEU metrics (BLEU-2 = 25.12, BLEU-3 = 11.85, BLEU-4 = 5.42) confirms its syntactic reasonableness and semantic coherence in generating complex traditional Chinese medicine (TCM) terminology and multi-word sequences. This advantage can be attributed to two key factors: (1) the XhTCM dataset, which integrates diverse, high-quality TCM-specific corpora, and (2) the LoRA fine-tuning strategy, which employs low-rank adaptation for computationally efficient domain specialization.

Comparative analysis reveals clear limitations of general-purpose language models in TCM-specific tasks. DeepSeek’s scores (39.63, 14.58, 6.84, and 3.03) outperform ChatGLM2-6B (26.44, 7.35, 4.06, and 1.37) but remain inferior to some TCM-specialized models. This suggests that general models lack training data tailored to TCM knowledge, resulting in reduced accuracy and fluency in domain-specific contexts.

Among TCM-specialized models, ShennongAlpha achieves the second-highest performance, leveraging its retrieval-augmented generation (RAG) architecture, which highlights the positive impact of knowledge retrieval mechanisms in TCM question-answering tasks. However, XuanHuGPT (LoRA) demonstrates superior adaptability to TCM-specific tasks, driven by the LoRA fine-tuning approach combined with the XhTCM dataset. In contrast, HuatuoGPT and MING-MOE exhibit relatively weaker performance, reflecting deficiencies in their training strategies for fine-grained TCM knowledge modeling.

The comparison between ChatGLM2-6B (P-Tuning v2) and XuanHuGPT (LoRA) provides insights into the efficacy of different parameter-efficient fine-tuning (PEFT) methods. XuanHuGPT’s LoRA approach outperforms P-Tuning v2, particularly on BLEU-2 (25.12 vs. 14.04) and BLEU-3 (11.85 vs. 7.34). This indicates that LoRA’s low-rank decomposition of weight updates enables more effective adaptation of the model’s internal representations to TCM-specific knowledge, surpassing the prompt-based optimization of P-Tuning v2 in terms of accuracy and fluency.

#### Performance evaluation on coverage metrics

In terms of model coverage (ROUGE) metrics, XuanHuGPT (LoRA) achieved the highest scores, with ROUGE-1: 39.98, ROUGE-2: 19.06, and ROUGE-L: 32.24. These results demonstrate that XuanHuGPT excels in capturing word-level content (ROUGE-1), bigram-level sequences (ROUGE-2), and maintaining structural coherence (ROUGE-L). This highlights the model’s ability to generate responses that retain both the semantic and structural integrity of Traditional Chinese Medicine (TCM)-related content, which is crucial for professional TCM consultations (Table 3).

**Table 3:** Evaluation results on ROUGE metrics

LLMs	ROUGE-1	ROUGE-2	ROUGE-L
DeepSeek	38.75	12.33	30.64
ChatGLM2-6B	33.32	12.37	24.29
HuatuoGPT	35.92	14.87	27.56
ShennongAlpha	39.19	13.60	30.65
MING-MOE	33.21	12.24	24.21
ChatGLM2-6B(P-Tuning v2)	38.28	14.71	28.37
**XuanHuGPT(LoRA)**	**39.98**	**19.06**	**32.24**

Among the TCM-specific models, ShennongAlpha ranked second with ROUGE-1: 39.19, ROUGE-2: 13.60, and ROUGE-L: 30.65, which are close to the performance of XuanHuGPT. Its retrieval-augmented generation (RAG) architecture enhances the retrieval capability of TCM knowledge, making it strong in content coverage.

Notably, DeepSeek's ROUGE-1 (38.75) and ROUGE-L (30.64) scores are very close to those of the specialized model ShennongAlpha, indicating its strong generalization ability in content coverage and structural preservation. This similarity may be attributed to DeepSeek’s Mixture of Experts (MoE) architecture and Multi-Head Latent Attention (MLA) mechanism, which enable it to capture a wide range of semantic information when processing complex texts. However, its ROUGE-2 score (12.33) is significantly lower than that of XuanHuGPT and some other specialized models, reflecting its limitations in capturing specific sequential patterns within the TCM domain. This may be due to DeepSeek’s pre-training data being primarily focused on general domains, without dedicated optimization for TCM terminology, prescription patterns, or clinical diagnoses. While its MoE architecture and MLA mechanism enhance its semantic expression, its specialization and ability to capture sequential content in the TCM field remain insufficient compared to XuanHuGPT and ShennongAlpha. This further underscores the importance of domain-specific fine-tuning (such as LoRA and RAG) in improving the model's adaptability to specialized tasks.

#### Performance evaluation on fluency/consistency/sensitivity metrics

Table 4 presents the performance of various large language models (LLMs) on the METEOR, BERTScore, and Embedding Distance metrics, which respectively evaluate semantic fluency, semantic consistency, and sensitivity to subtle input variations.

**Table 4:** Evaluation results on METEOR, BERTScore, and embedding distance metrics

LLMs	METEOR	BERTScore	Embedding Distance
DeepSeek	0.105	0.776	0.862
ChatGLM2-6B	0.174	0.775	0.854
HuatuoGPT	0.220	0.776	0.861
ShennongAlpha	0.149	**0.892**	0.829
MING-MOE	0.218	0.738	0.839
ChatGLM2-6B(P-Tuning v2)	0.185	0.850	0.866
**XuanHuGPT(LoRA)**	**0.260**	0.868	**0.870**

XuanHuGPT (LoRA) continues to demonstrate strong performance across all three metrics, indicating its capability to generate fluent, semantically coherent, and context-sensitive responses tailored to Traditional Chinese Medicine (TCM) tasks. For TCM-specific LLMs, semantic consistency and sensitivity are critical for practical implementation in clinical decision support systems, underscoring their real-world significance.

ChatGLM2-6B (P-Tuning v2) outperforms DeepSeek in both METEOR (0.185) and BERTScore (0.850), although it still falls short of XuanHuGPT (LoRA). DeepSeek shows competitive results in BERTScore and Embedding Distance, suggesting its potential as a general-purpose model. However, its lower METEOR score reflects limitations in generating fluent and semantically appropriate responses for domain-specific TCM tasks.

The domain-specific TCM model ShennongAlpha achieves a notably high BERTScore (0.892), highlighting the effectiveness of its Retrieval-Augmented Generation (RAG) architecture in retrieving relevant information from TCM literature and enhancing semantic consistency. Nevertheless, its relatively low METEOR (0.149) and Embedding Distance (0.829) scores suggest limited ability in producing fluent and coherent responses, as well as in capturing subtle variations in input, such as nuanced symptom descriptions.。

#### Multi-dimensional comprehensive evaluation

**Fig. 4:**
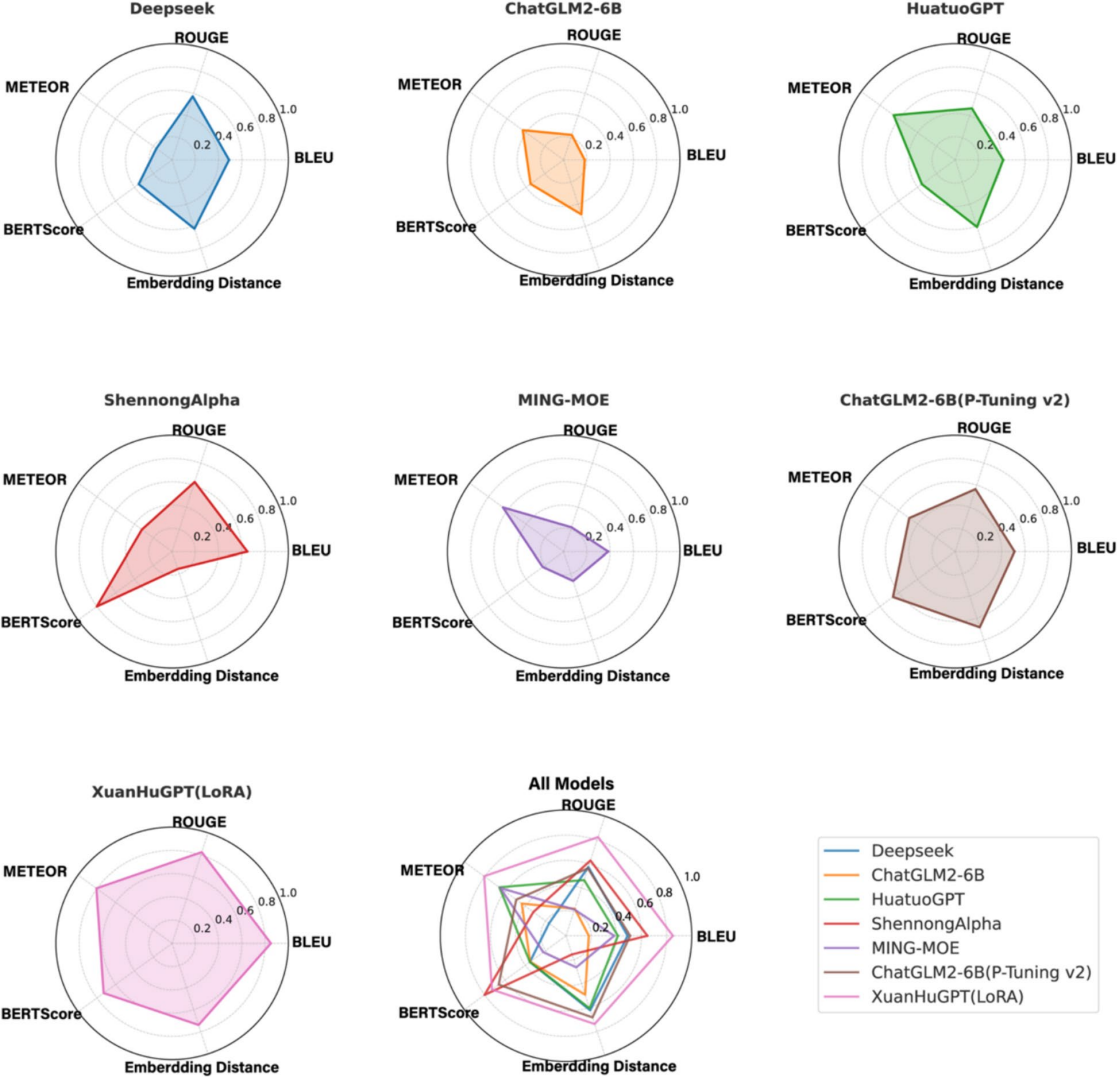
Radar chart of models performance comparison

We employed a “Z-score + Sigmoid” approach to normalize the five evaluation metrics. This method combines Z-score standardization with a Sigmoid transformation, preserving the scaling benefits of Z-score normalization while compressing extreme values through the Sigmoid function, thereby enhancing robustness. The normalized results are presented in Table 5. Furthermore, to better visualize the overall performance of the models, a radar chart is plotted, as shown in Fig. 4.

**Table 5:** Z-score + Sigmoid normalized LLM metrics

LLMs	BLEU (accuracy)	ROUGE (coverage)	METEOR (fluency)	BERTScore (consistency)	Embedding distance (sensitivity)
DeepSeek	0.490	0.575	0.167	0.355	0.623
ChatGLM2-6B	0.181	0.226	0.435	0.352	0.493
HuatuoGPT	0.413	0.466	0.655	0.355	0.607
ShennongAlpha	0.647	0.629	0.321	0.801	0.156
MING-MOE	0.383	0.219	0.646	0.223	0.264
ChatGLM2-6B (P-Tuning v2)	0.510	0.564	0.489	0.662	0.683
XuanHuGPT(LoRA)	0.850	0.825	0.805	0.728	0.738

XuanHuGPT (LoRA) demonstrates the largest radar chart area with a shape approximating a regular pentagon, indicating consistently strong performance across all five metrics and the best overall capability. Its high scores in BLEU, ROUGE, and METEOR highlight its advantages in generating accurate, comprehensive, and fluent TCM texts. The high score in Embedding Distance further supports its sensitivity to subtle semantic variations.

ShennongAlpha shows a prominent peak in BERTScore, but significant contraction in METEOR and Embedding Distance, suggesting imbalanced performance—strong in semantic consistency but weaker in fluency and sensitivity. ChatGLM2-6B (P-Tuning v2) displays a more balanced radar shape, albeit with a smaller area, indicating that while it outperforms general-purpose models, it still lags behind XuanHuGPT (LoRA) on TCM-specific tasks. HuatuoGPT and MING-MOE exhibit relatively better performance in METEOR, but their radar charts are smaller and less comprehensive overall, reflecting limited general capability in TCM tasks. DeepSeek and ChatGLM2-6B (in general settings) present the smallest and most irregular radar chart areas, indicating relatively poor performance in TCM-specific tasks—especially in terms of fluency and semantic consistency.

#### Performance evaluation on safety

XuanHuGPT (LoRA) and baseline models were evaluated for safety using identical TCM-related questions from a shared test set. GPT-4o initially assessed responses across three safety dimensions, with scores validated by domain experts. Outcomes were classified as “Win,” “Tie,” or “Loss,” based on the models’ ability to deliver safe, accurate, and ethically sound TCM responses.

XuanHuGPT (LoRA) outperformed general-purpose models like DeepSeek and ChatGLM2-6B, achieving high win rates (25%) and low loss rates (16.0%–17.0%). This advantage stems from LoRA fine-tuning on the XhTCM dataset, integrating classical TCM literature with modern pharmacology, enabling precise handling of TCM terminology, pathogenesis, and treatment strategies while minimizing overgeneralization and inaccuracies.

Compared to ShennongAlpha, XuanHuGPT (LoRA) showed comparable safety performance, with balanced win and loss rates (25.0% each) and a high tie rate (50.0%). Against other TCM-specific models, it achieved superior win rates: 80.0% versus MING-MOE, 45.0% versus HuatuoGPT, and 41.0% versus ChatGLM2-6B (P-Tuning v2).

**Fig. 5:**
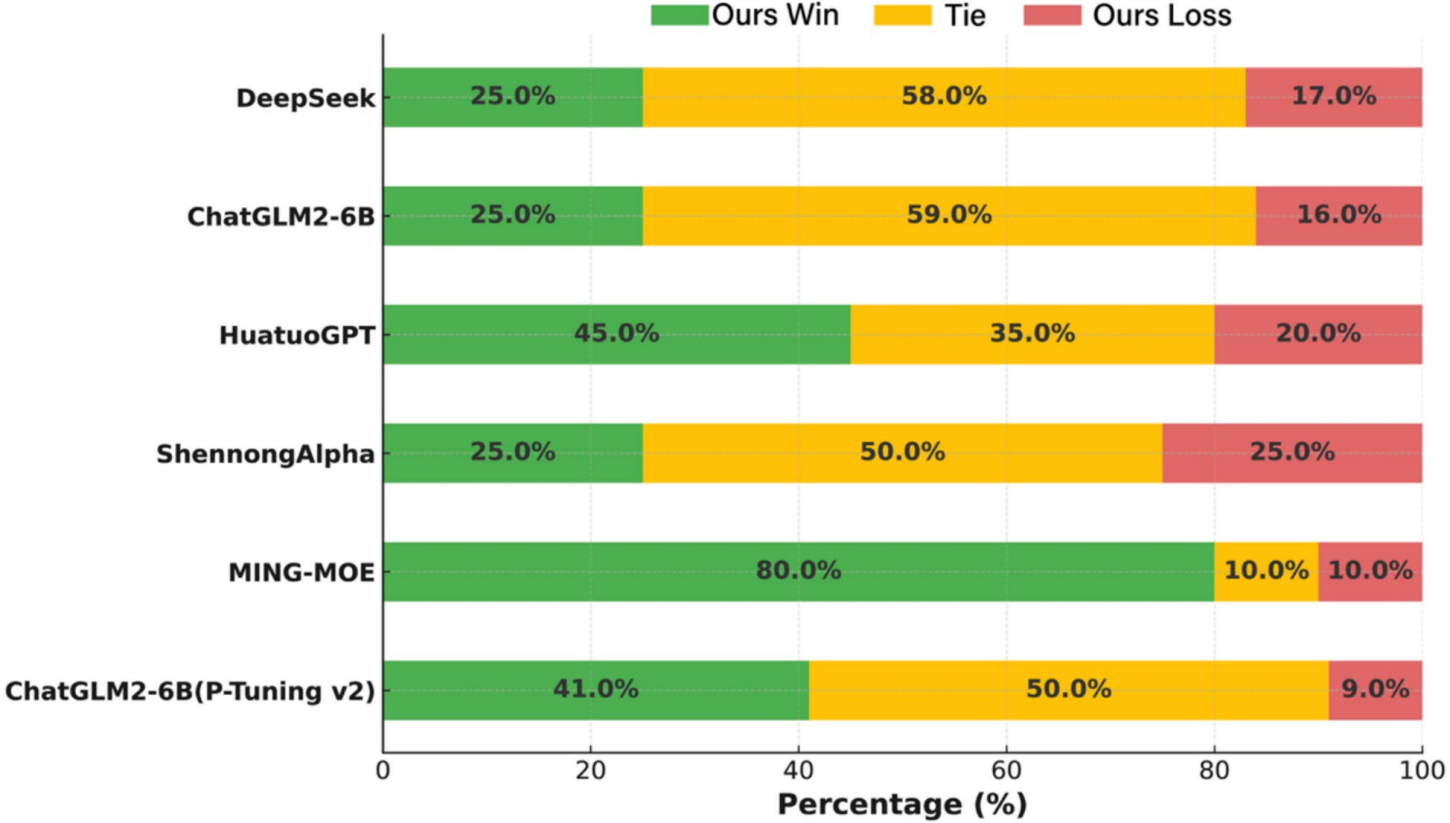
Evaluation results for safety

XuanHuGPT (LoRA) consistently exhibited low loss rates, reflecting robust safety in TCM contexts. Its ability to perform syndrome differentiation and formulate treatment plans using authentic TCM logic underscores its professional depth and clinical applicability as a specialized TCM language model (Fig. 5).

#### Model architecture and fine-tuning method analysis

Model performance differences primarily stem from fine-tuning methods, architectural design, and dataset specificity.

1. Differences between general-purpose and domain-specific fine-tuning.

General-purpose models (e.g., DeepSeek and ChatGLM2-6B) are pretrained on broad-domain data, lacking specialized Traditional Chinese Medicine (TCM) content, making it challenging to deeply understand TCM terminology, disease classification, and treatment methods. Domain-specific models (e.g., XuanHuGPT(LoRA) and ShennongAlpha) significantly enhance TCM task performance through targeted fine-tuning on TCM knowledge. XuanHuGPT(LoRA) leverages the XhTCM dataset, integrating classical TCM literature, clinical knowledge, and pharmacological information, ensuring precise mastery of TCM theory and applications.

(2) Comparison of fine-tuning methods

LoRA (XuanHuGPT(LoRA)) reduces storage demands via low-rank matrix decomposition, adjusting only newly added parameters to efficiently incorporate TCM knowledge while maintaining computational efficiency. Its strength lies in significantly improving adaptability to TCM tasks, particularly in scenarios requiring deep domain expertise. In contrast, P-Tuning v2 (ChatGLM2-6B(P-Tuning v2)) enhances task adaptability by optimizing prompt embeddings but focuses on prompt adjustments, struggling to capture intricate TCM details, resulting in inferior performance compared to LoRA.

(3) Differences between RAG and MoE architectures

ShennongAlpha employs a Retrieval-Augmented Generation (RAG) architecture, which retrieves and integrates external knowledge into the generation process, enhancing content accuracy and semantic consistency, particularly for specialized TCM tasks. DeepSeek uses a Mixture of Experts (MoE) architecture, dynamically selecting expert models to improve computational efficiency. However, its domain adaptability is limited, lacking deep understanding of TCM terminology and treatment protocols, leading to suboptimal performance in TCM tasks compared to specialized models.

(4) Knowledge integration and dataset differences

The XhTCM dataset, designed specifically for TCM tasks, integrates authoritative TCM resources, providing robust knowledge support for XuanHuGPT(LoRA). This enables superior performance across multiple metrics, generating fluent and professionally accurate TCM responses. In contrast, DeepSeek’s dataset focuses on general domains, lacking specialized TCM training, resulting in poorer performance in TCM-specific tasks.

### Qualitative analysis

To more accurately evaluate model performance in practical Traditional Chinese Medicine (TCM) scenarios, we designed four representative tasks: herbal inquiry, symptom analysis, disease treatment, and prescription evaluation, corresponding to Appendix Tables 6, 7, 8, 9. Each scenario includes an expert-validated reference answer as a benchmark to assess the models’ capabilities in terms of professional accuracy, logical coherence, and clinical relevance.

#### Herbal inquiry

*Case of Schizonepetae Carbonisatus (Appendix *Table 6*)* In this task, XuanHuGPT (LoRA) was able to systematically describe the therapeutic indications, pharmacological mechanisms, usage methods, and contraindications of *Schizonepetae Carbonisatus*. Its response covered a wide range of relevant knowledge with accurate expression and terminology, closely aligning with classical TCM literature and expert reference standards. The model not only mentioned common effects such as “hemostasis,” “dispelling wind,” and “clearing heat,” but also clearly articulated appropriate applications and usage cautions—demonstrating both high-quality text generation and robust understanding of TCM-specific vocabulary.

In contrast, DeepSeek displayed partial understanding of the herb’s processing methods and clinical indications, but lacked structural organization and reasoning. ShennongAlpha, benefiting from its RAG architecture, produced relatively authoritative content, though the responses tended to be rigid and linguistically repetitive. Other models—such as MING-MOE, HuatuoGPT, and ChatGLM2-6B—exhibited various degrees of deviation or generalization in describing pharmacological actions, and occasionally misused technical terms or misunderstood herbal properties. Overall, XuanHuGPT was the only model that consistently demonstrated reliable comprehension and professional expression in the domain of herbal knowledge.

#### Symptom analysis

*Case of damp-phlegm type functional dyspepsia (Appendix *Table 7*)* This task emphasized the models’ capacity for syndrome differentiation based on clinical symptoms. XuanHuGPT accurately identified the core pathogenesis of “spleen deficiency with dampness encumbrance” and proposed a treatment principle centered on “transforming dampness, harmonizing the middle, regulating Qi, and relieving nausea.” It also recommended commonly used formulas such as *Er Chen Tang* and *Huo Xiang Zheng Qi San*, reflecting a reasonable line of thought and a certain level of clinical applicability.

Among other models, DeepSeek partially recognized the symptoms’ diagnostic category, but its explanation of pathogenesis was vague and lacked clear therapeutic prioritization. ShennongAlpha listed multiple herbal components but failed to justify their selection within a coherent diagnostic framework. ChatGLM2-6B and its P-Tuning v2 variant provided general descriptions without articulating the principle of “pattern differentiation and treatment.” Meanwhile, HuatuoGPT and MING-MOE tended to focus on lifestyle advice and offered health education-style content, with insufficient grounding in TCM theory.

In summary, XuanHuGPT showed relatively robust performance in symptom attribution, pattern diagnosis, and formula selection, better aligning with the reasoning process required in clinical TCM contexts.

#### Disease treatment

*Case of prescription design for gouty nephropathy (Appendix *Table 8*)* This scenario evaluated the models’ ability to design treatment strategies tailored to specific disease patterns. XuanHuGPT combined pathogenesis concepts such as “damp-heat accumulation in the lower burner” and “kidney deficiency with dampness” to propose a dual-treatment approach. The suggested formulas—including *Zhi Bai Di Huang Wan* and *Bi Xie Fen Qing Yin*—were appropriately selected, and the rationale behind the composition was logically presented, consistent with core TCM treatment principles.

ShennongAlpha provided a detailed list of herbs, but its treatment plan lacked a foundation in syndrome differentiation and failed to establish a link between pathogenesis and formula selection. DeepSeek’s solution was brief and superficial, with limited differentiation between disease subtypes and few modification strategies. MING-MOE and HuatuoGPT mainly adopted modern medical concepts such as “kidney function regulation” and “diuresis,” with limited application of layered TCM logic. ChatGLM2-6B generated only generic descriptions, reflecting a lack of domain-specific reasoning.

XuanHuGPT demonstrated a relatively strong ability in disease mechanism interpretation and prescription logic. While there is still room to further standardize its expression, the model generally outperformed others in both plausibility and domain-specific accuracy.

#### Prescription evaluation

*Case of pediatric asthma due to kidney Qi deficiency (Appendix *Table 9*)* This task assessed the models’ capability to adjust existing formulas based on individual constitution and syndrome differentiation. XuanHuGPT accurately identified the core pattern of “deficiency of kidney Qi causing wheezing” and provided targeted suggestions for herb additions and subtractions. Its recommendations reflected an awareness of pediatric physiological characteristics and associated contraindications, indicating a moderate level of clinical reasoning and customization.

Although ShennongAlpha proposed several herbal options, it failed to clearly relate them to the identified pattern. DeepSeek’s logic was weak, with inconsistencies in the properties of the suggested herbs. Other models—including ChatGLM2-6B, MING-MOE, and HuatuoGPT—mostly repeated standard formulas without demonstrating individualized modification based on syndrome presentation.

XuanHuGPT showed the ability to tailor formulas in response to syndrome-specific insights, reflecting a higher level of task adaptability and basic diagnostic reasoning. However, its performance in handling more complex prescriptions and patient variability still requires further refinement.

## Conclusion and limitations

This paper introduces XuanHuGPT, an AI-based large language model specifically designed for intelligent Traditional Chinese Medicine (TCM) diagnosis. The model supports TCM-related Q&A, enhances diagnostic and treatment decisions, and aids in health management and prevention.

By utilizing the XhTCM dataset—a comprehensive and high-quality corpus that integrates authoritative TCM resources such as ShenNong_TCM_Dataset, TCMBank, and TCMIP v2.0—XuanHuGPT provides precise, contextually relevant answers to inquiries on TCM theories, clinical practices, herbal pharmacology, and prescription formulations. The model is optimized using the parameter-efficient fine-tuning (PEFT) technique, specifically LoRA and P-Tuning v2, based on the ChatGLM2-6B base model, achieving a balance between computational efficiency and domain-specific performance. By establishing a comprehensive evaluation framework, which includes quantitative metrics like BLEU, ROUGE, METEOR, BERTScore, and Embedding Distance, as well as expert qualitative assessments, XuanHuGPT significantly outperforms general-purpose models and other TCM-specific models in terms of accuracy, coverage, fluency, consistency, sensitivity, and safety. This study establishes a robust framework for TCM-specific large language models, contributing to the digitalization, intelligent dissemination, and global promotion of TCM knowledge.

Despite these advances, XuanHuGPT has several limitations. The training process was constrained by hardware resources, and the relatively lightweight architecture of the base model may limit its performance in highly specialized TCM subfields. Additionally, while XuanHuGPT provides valuable insights, its outputs should not replace professional medical advice, and validation by TCM practitioners or healthcare professionals is essential for clinical applications. The model's reliance on structured data may also limit its ability to handle unstructured or multimodal inputs, such as real-time diagnostic data.

Future work will focus on integrating the four diagnostic methods of TCM (observation, listening and smelling, questioning, and pulse-taking) to construct a more comprehensive multimodal TCM dataset, further enhancing the capabilities of XuanHuGPT. This will enable the model to process diverse data types and provide more comprehensive advisory services.

## Data Availability

The data used to support the findings of this study are included within the paper.

## Appendix A: Model performance in practical TCM scenarios

In the qualitative analysis, this study selected four aspects for evaluation: medicinal herbs, symptoms, and diseases, prescription evaluation, to assess the professionalism of each model in answering questions related to Traditional Chinese Medicine (TCM) knowledge. The specific information regarding each model’s responses to questions about medicinal herbs is presented in Appendix Table 6. The specific information regarding responses to questions about symptoms is presented in Appendix Table 7. The specific information regarding responses to questions about diseases is presented in Appendix Table 8.The specific information regarding responses to questions about prescription evaluation is presented in Appendix Table 9.

### Inquiry on herbs

Functions and uses of schizonepeta charcoal? (荆芥炭的功效与作用?).

**Table 6:** Inquiry on herbs

LLMs	Response
MING-MOE	荆芥炭是一种常见的中药材, 具有炭化后形成的黑色炭质, 有清热解毒、润肺止咳、止血消炎、镇痛止痒等功效。下面是荆芥炭的功效和用法: 1. 清热解毒: 荆芥炭具有清热解毒的作用, 可以帮助清除体内的热毒, 缓解喉咙疼痛、口腔溃疡等症状。 2. 润肺止咳: 荆芥炭可以刺激呼吸道, 增加黏液分泌, 缓解咳嗽、喉咙不适等症状。 3. 止血消炎: 荆芥炭可以收敛伤口, 促进伤口愈合, 还可以用于治疗创伤、感染、炎症等疾病。 4. 镇痛止痒: 荆芥炭可以镇痛、止痒, 适用于缓解刀枪不入、皮肤破裂、瘙痒等症状。 用法: 1. 饮用: 将荆芥炭加入开水中, 饮用即可。 2. 炖汤: 将荆芥炭和其他食材一起炖汤, 具有调味、营养的作用。 3. 煎药: 将荆芥炭加入药锅中, 加水煎至预定剂量。 4. 泡脚: 将荆芥炭加入温水中, 泡脚15分钟, 可缓解脚部疲劳、缓解疼痛。 5. 涂抹: 将荆芥炭制成膏状, 涂抹在伤口、皮肤破裂等部位。 荆芥炭虽然功效显著, 但也应注意适量使用, 避免过量使用导致不良反应。同时, 对于孕妇、儿童、老年人等特殊人群, 应在医生指导下使用。
HuatuoGPT	荆芥炭 (科学名: Synedra Chinensis) 是一种常见的中药材, 属于蒺藜科荆芥属植物。荆芥炭具有凉血止血、散瘀止痛的功效。 荆芥炭的主要药效成分是荆芥炭素, 具有很好的止血作用, 能够收敛血管, 减少血管通透性, 从而达到止血的效果。因此, 荆芥炭常用于临床治疗各种出血症状, 如吐血、衄血、便血、崩漏等。 此外, 荆芥炭还具有散瘀止痛的作用。它的药性偏热, 能够活血化瘀, 消除瘀血阻滞, 从而缓解疼痛。因此, 荆芥炭也常用于治疗各种疼痛症状, 如瘀血痛经、跌打损伤等。 荆芥炭的用法主要是煎汤内服。一般情况下, 成人每次服用3-9克, 煎汤内服。在服用荆芥炭时, 应注意遵医嘱, 不可随意增减药量。对于过敏荆芥炭的人, 应避免使用。 需要注意的是, 虽然荆芥炭具有很好的止血和散瘀止痛的作用, 但并不是所有的出血症状都适合使用荆芥炭来治疗。例如, 由于荆芥炭性温燥, 热盛血热、阴虚火旺的人应慎用。此外, 荆芥炭也有一定的副作用, 如用药过量可能会导致胃肠道不适等症状。因此, 在使用荆芥炭时, 最好在医生的指导下进行。
ChatGLM2-6B	荆芥炭是一种中药材,具有祛风止血、温中止呕的功效。常用于治疗血虚血旺、月经过多、崩漏、吐血、衄血、咯血、尿血、便血、虚寒腹痛、胃寒呕吐、泄泻、疮肿、跌打肿痛等症状。 荆芥炭的用法一般是将其点燃,制成炭,然后外用或内服。外用时,可以将其制成炭外敷,或者将其研末撒布于患处。内服时,可以将其煎汤或泡茶饮用。不过,荆芥炭的性质较为温热,所以在使用时需要根据具体病情来合理使用。
ChatGLM2-6B (P-Tuning v2)	荆芥炭是一种中药材, 具有清热解毒、祛风止痛、活血化瘀等功效。常用于治疗感冒、头痛、咳嗽、胃痛、疮疖等症状。用法方面, 可以煎服, 每次用量1-2克, 每日3-4次。同时, 也可以外用, 将荆芥炭研成粉末, 涂抹于牙龈出血处, 或者将荆芥炭撒于牙龈上, 轻压使其吸收。 需要注意的是, 荆芥炭孕妇慎用, 不宜过量使用。同时, 在使用荆芥炭时, 应遵循医嘱或医师的建议。
Shengnong- Alpha	荆芥炭 (nmm-01da) 是中药材荆芥经过特殊炮制而成的一种加工品, 其主要功效和用法如下: 1、功效 荆芥炭具有止血、凉血、散瘀的作用。它常用于治疗出血性疾病, 如吐血、衄血、便血、崩漏等。此外, 荆芥炭还能清热解毒, 对于一些因热毒引起的症状也有一定的辅助疗效。 2、用法 荆芥炭通常以煎汤内服的方式使用, 也可以与其他中药配伍使用。具体的用量应根据医生的建议来确定, 一般常用剂量为3 ~ 9克。 3、注意事项 荆芥炭性味辛、微苦、微温, 体质偏寒者应慎用。 使用前最好咨询中医师或药师, 以确保安全有效。
DeepSeek	荆芥炭是中药荆芥的炮制品, 由荆芥经炒炭 (高温煅烧至表面焦黑、内部焦黄) 制成, 其功效和应用与生荆芥有所不同。以下是其具体功效和用法: 1、功效 1. 收敛止血: 荆芥炭主要适用于各种出血证, 如吐血、衄血 (鼻出血) 、便血、崩漏 (子宫出血) 等, 通过炭药的收敛作用达到止血效果。 2. 祛风解表 (较弱): 保留荆芥生品部分祛风散寒的功效, 但作用较弱, 更侧重于止血。 3. 散瘀消疮: 用于外伤出血或皮肤疮疡, 可外用或内服。 2、适用症状 妇科: 月经过多、崩漏。内科: 消化道出血、痔疮出血。外科: 外伤出血 (可研末外敷) 。 3、用法与用量 1. 内服: 煎汤: 3 ~ 10克, 宜包煎 (避免药液浑浊) 。 研末冲服: 1 ~ 3克, 止血效果更佳。 2. 外用: 研细粉撒敷患处, 用于外伤或溃疡出血。 4、注意事项 1. 辨证使用: 适用于血热或虚寒性出血, 但需配伍其他药物 (如血热配生地、侧柏叶; 虚寒配炮姜、艾叶) 。 2. 禁忌: 表证 (感冒) 无出血者慎用, 以免收敛邪气。孕妇慎用, 需遵医嘱。 3. 储存: 密封防潮, 避免失效。 5、常见配伍 配侧柏叶、茜草: 增强凉血止血作用。 配当归、艾叶: 调理妇科虚寒性出血。 建议在中医师指导下使用, 确保对症安全。
XuanHuGPT (LoRA)	荆芥炭是一种中药材, 具有清热解毒、祛风止血的功效。常用于治疗头痛、牙痛、口腔溃疡、皮肤瘙痒、烧伤、吐血、衄血、咯血、崩漏等症状。 荆芥炭具有止血、祛风、清热、解毒的作用, 主要用于治疗上述疾病。荆芥炭可以促进伤口愈合,止血效果显著,对皮肤瘙痒、口腔溃疡等疾病也具有良好的疗效。 荆芥炭的用法一般为粉末冲服,或者外用适量,研末调敷。用量要根据病情而定,一般每次用量在10克左右。荆芥炭不适合孕妇、哺乳期妇女和儿童使用,也不宜与某些药物同用。在使用过程中,如出现不良反应,应立即停止使用,并咨询医生。
Reference Answer: TCM Experts[41]	为唇形科植物荆芥的全草。 主治感冒发热, 头痛, 咽喉肿痛, 中风口噤, 吐血, 衄血, 便血; 崩漏, 产后血晕; 痈肿, 疮疥, 瘰疬。荆芥穗效用相同, 惟发散之力较强。 用法用量: 内服: 煎汤, 3 ~ 10 g; 或临丸、散。 外用: 适量, 煎水熏洗; 捣敷; 或研末调散。

### Inquiry on symptoms

The patient presents with chest and abdominal stuffiness, a feeling of heaviness in the body, dizziness, poor appetite, hiccupping accompanied by nausea and vomiting, a tasteless mouth without thirst, and a thick greasy tongue coating among other symptoms. Please provide a treatment method.

(患者表现为胸腹痞满、身体沉重、头晕、食欲不振、嗳气伴恶心呕吐、口中寡味而不渴, 舌苔厚腻等症状。请提供治疗方法。).

**Table 7:** Inquiry on symptoms

LLMs	Response
MING-MOE	这些症状可能是由多种原因引起的, 需要根据具体情况来确定治疗方法。以下是一些常见的治疗方法和建议: 1. 休息和饮食调整: 如果感到身体沉重, 头晕, 食欲不振, 打嗝, 恶心和呕吐等症状, 可能是因为身体过度劳累或饮食不当引起的。建议患者多休息, 饮食要清淡易消化, 避免过度劳累和暴饮暴食。 2. 中药调理: 中药可以通过调节身体的阴阳平衡来缓解这些症状。建议患者咨询中医师, 根据个人体质和症状开具合适的中药处方。 3. 针灸: 针灸可以通过刺激人体的穴位来调节身体的功能, 缓解这些症状。建议患者在中医师的指导下进行针灸治疗。 4. 西医治疗: 如果以上方法不能缓解症状, 可以考虑进行西医治疗。可能的治疗方法包括药物治疗、手术治疗等。在使用药物治疗时, 需要根据患者的具体症状和病情来选择药物。 5. 健康生活方式: 保持健康的生活方式也有助于缓解这些症状。建议患者戒烟限酒, 保持充足的睡眠, 饮食均衡, 积极参加体育锻炼等。 请注意, 以上建议仅供参考。如果症状严重或持续时间较长, 建议尽快就医, 以便进行更专业的诊断和治疗。
HuatuoGPT	患者胸部和腹部闷、身体感到沉重、头晕、食欲不振、打嗝、伴有恶心和呕吐、口中无味但不渴、舌苔厚腻的一系列症状可能是由湿气和痰湿内阻引起的。在中医中, 这种情况通常被称为"湿痹"或"痰湿"。以下是一些可能的中医治疗方案: 1.去痰湿: 藿香正气水有祛痰、理气的功效, 适用于治疗因湿痹引起的胸口、呕吐等症状。 2.健脾化湿: 六君子汤有健脾益气、燥湿化痰的功效, 适用于治疗因湿痹引起的胸口、呕吐等症状。 3.清热利湿: 三石汤有清热利湿、和胃止呕的功效, 适用于治疗因湿痹引起的胸口、呕吐等症状。 4.健脾和胃: 二陈汤有健脾和胃、燥湿化痰的功效, 适用于治疗因湿痹引起的胸口、呕吐等症状。 请注意, 以上的方剂和剂量需要由专业的中医师根据您的具体情况配制和调整。此外, 饮食方面, 应避免油腻、甜食和冷饮, 多吃新鲜蔬菜和水果, 保持良好的生活习惯, 如适量锻炼和充足休息, 也有助于改善症状。
ChatGLM2-6B	根据您描述的症状, 可能是脾胃虚弱、气滞、痰湿等病症引起的。以下是一些建议供您参考: 饮食习惯调整: 避免食用辛辣、油腻、刺激性食物, 多吃清淡易消化的食物, 如粥、面、蔬菜等。 保持心情舒畅: 避免情绪波动, 多参与轻松的活动, 如散步、瑜伽、听音乐等。 适当的运动: 进行适量的有氧运动, 如散步、慢跑等, 有助于改善症状。 中药治疗: 中医认为此症状可能是脾胃虚弱、气滞、痰湿所致, 可以考虑服用一些中药进行治疗。具体药物及剂量应根据您的实际情况和中医师的建议进行。 针灸疗法: 通过针刺穴位, 如足三里、关元等穴位, 可以改善症状。针灸疗法需要专业医师操作, 建议您在医生的指导下进行。 推拿按摩: 通过按摩腹部及穴位, 可以缓解症状, 但需要注意按摩手法和穴位选择, 避免自行操作。 寻求医生帮助: 如症状持续或加重, 建议您及时就医, 寻求专业医生的帮助进行诊断和治疗。请注意, 以上建议仅供参考, 具体治疗方案还需根据您的实际情况和医生建议进行。
ChatGLM2-6B (P-Tuning v2)	根据您的描述, 患者可能患有脾胃湿滞、气滞胃肠道等症状。以下是几种可能的治疗方法: 1. 脾胃湿滞: 可以尝试使用陈皮、半夏、茯苓等中药材煮汤, 或者使用砂仁、草果等物品煮粥。这些中药都有健脾、化湿、理气的功效, 可以帮助改善脾胃湿滞的情况。 2. 气滞胃肠道: 可以尝试使用紫苏、薄荷、黄连等中药材煮汤, 或者使用木香、郁李仁等物品煎服。这些中药都有行气、解郁、清热、解毒的功效, 可以帮助缓解气滞胃肠道的不适症状。 需要注意的是, 中药治疗需要在医生指导下进行, 避免自行使用。同时, 饮食也需要注意清淡、易消化, 避免油腻、辛辣等刺激性食物。
Shengnong-Alpha	根据您描述的症状, 这些症状在中医中通常被认为是湿邪困脾或痰湿内阻的表现。治疗应以健脾化湿、理气和胃为主。以下是一些可能的治疗方法和建议: 1、中药治疗 阿胶 (nmm-015 s): 具有补血滋阴、润燥止血的功效, 适用于气血不足、虚劳咳嗽、吐血等症状。对于湿邪困脾引起的症状, 可配合其他药物使用。 鹗骨 (nmm-06eu): 具有续筋骨、消肿痛的作用, 常用于跌打损伤、骨折等。虽然它主要用于外伤, 但若患者因湿邪导致身体沉重、关节不适, 也可以考虑配合使用。 栗壳 (nmm-031j): 具有降逆生津、化痰止咳、清热散结、止血等功效, 适用于反胃、呕哕、咳嗽痰多等症状。 建议在专业中医师的指导下, 根据具体症状选择合适的中药配方。 2、饮食调理 避免食用油腻、甜食、冷饮等容易加重湿邪的食物。 多吃一些有助于健脾化湿的食物, 如山药、薏米、茯苓、莲子等。 可以适量饮用生姜茶, 帮助温中散寒、缓解恶心和呕吐。 3、生活方式调整 保持规律的作息时间, 避免过度劳累。 适当进行锻炼, 如散步、太极等, 有助于改善脾胃功能。 注意保暖, 尤其是腹部和背部, 避免湿气侵入体内。 4、针灸与推拿 针灸可以通过刺激穴位 (如足三里、中脘、内关等) 来调节脾胃功能, 缓解胸闷、腹胀、恶心等症状。 推拿可以帮助疏通经络, 促进气血运行, 缓解身体沉重感。
DeepSeek	根据患者症状 (胸腹部闷胀、身重头昏、食欲不振、嗳气恶心呕吐、口淡不渴、舌苔厚腻), 辨证为湿浊中阻, 脾胃气滞, 治疗应以 健脾化湿、理气和胃 为主。以下是具体治疗方案: 1. 中药方剂 推荐方: 藿香正气散加减 (偏寒湿) 或 三仁汤加减 (偏湿热) 基础组方: - 藿香 10 g (芳香化湿、和胃止呕) - 苍术 10 g (燥湿健脾) - 厚朴 10 g (行气除满) - 半夏 9 g (降逆止呕) - 茯苓 15 g (健脾利湿) - 陈皮 10 g (理气化痰) - 白蔻仁 6 g (化湿行气) - 薏苡仁 20 g (渗湿健脾) - 生姜 3片 (温中止呕) 加减: - 若舌苔黄腻、口苦, 加 黄连 3 g、竹茹 10 g (清热化湿); - 若腹胀明显, 加 木香 6 g、大腹皮 10 g (行气消胀); - 若头昏重, 加 石菖蒲 10 g、佩兰 10 g (化湿醒神) 。 2. 中成药选择 - 藿香正气水/胶囊 (寒湿型, 恶心身重明显); - 保和丸 (食积伴嗳腐酸臭); - 香砂六君丸 (脾虚湿阻长期调理) 。 3. 外治法 - 艾灸: 中脘、足三里、阴陵泉 (温阳化湿); - 刮痧: 背部膀胱经 (脾俞、胃俞) 或腹部 (中脘 → 气海), 助湿浊外泄。 4. 饮食调理 - 忌口: 生冷、油腻、甜食、乳制品 (助湿生痰); - 食疗方: - 陈皮薏米粥 (陈皮5g + 薏苡仁30g + 大米50g, 煮粥); - 紫苏生姜饮 (紫苏叶5g + 生姜3片, 煎水代茶) 。 5. 生活调护 - 避免久处潮湿环境, 适当运动 (如八段锦 “调理脾胃须单举”); - 睡前顺时针摩腹5分钟 (助运化) 。 辨证要点 若患者呕吐物清稀、舌苔白腻, 属 寒湿, 需加强温化 (如加干姜); 若呕吐酸苦、苔黄腻, 属 湿热, 需加黄连、竹茹。建议结合脉象 (濡缓或滑数) 进一步判断。 注: 若症状持续或加重, 需排除器质性疾病 (如胃炎、肝胆疾病), 建议就医检查。
XuanHuGPT (LoRA)	根据症状描述, 患者可能患有湿痰证。根据中医理论, 湿痰证的病机为湿痰内停, 中气不足, 脾胃失运, 气机不和。治疗需要健脾化湿、理气降逆。 以下是治疗方法: 中药治疗: 苍术: 10克; 厚朴: 10克; 半夏: 10克; 陈皮: 10克; 紫苏梗: 10克; 桔梗: 10克; 藿香: 10克; 茯苓: 10克; 甘草: 6克; 生姜: 10克; 大枣: 4枚 2. 饮食调理: 饮食有节, 避免辛辣油腻食物; 多吃健脾利水之品 3. 针灸治疗: 上脘、中脘、下脘、足三里、天枢、内关、气海: 用毫针刺之, 留针30分钟 4. 推拿治疗: 推胸、腹部、背部, 以顺气为主 5. 艾灸治疗: 选用中脘、关元、气海等穴位, 进行温和的艾灸, 每次30分钟, 每周3次, 共4周 6. 拔罐治疗: 在腹部、背部等部位进行拔罐, 留罐10-15分钟, 每周3次, 共4周 请注意, 以上治疗方法仅供参考, 具体治疗方案需要根据您的症状、病程和病因进行调整。在使用中药之前, 建议咨询中医师或医生的意见, 并按照医生建议进行治疗。同时, 饮食有节, 调畅情志, 起居有时也是治疗湿痰证的重要方面。
Reference Answer: TCM Experts[42]	功能性消化不良 (痰湿中阻类): 主要症状: 脘腹痞塞不舒, 胸膈满闷, 身重困倦, 头昏纳呆, 嗳气呕恶, 口淡不渴, 舌苔厚腻, 脉沉滑。证机: 痰湿阻滞, 脾失健运, 气机不和。治法: 除湿化痰, 理气和中; 方以平胃散合二陈汤加减 (苍术、厚朴、半夏、陈皮、紫苏梗、桔梗、藿香等) 。中药服法: 每日1剂, 水煎取汁 400 mL, 分早晚 2次温服; 饮食有节, 调畅情志, 起居有时。 针刺治疗: 给予 “老十针”针刺治疗, 取穴上脘、中脘、下脘、足三里、天枢、内关、气海, 留针 30 min, 治疗 2 周 (每周 5 次) 。 灸法治疗: 隔药饼脐灸, 隔日治疗1次, 每周3次, 疗程4周。 推拿治疗、耳穴压豆治疗、穴位贴敷治疗、穴位埋线治疗、中医内外治法联合治疗

### Inquiry on diseases

What are the TCM prescriptions for treating gouty nephropathy?

**Table 8:** Inquiry on diseases

LLMs	Response
MING-MOE	痛风性肾病是痛风引起的肾脏疾病, 常常表现为肾脏红肿、疼痛、尿频、尿急等症状。中医药治疗痛风性肾病的方法包括以下几种: 1. 中药治疗: 中药治疗痛风性肾病是比较常见的方法。中药配方通常包括清热利湿、祛风解毒、益气固表、滋补肝肾等功效。中医师可以根据患者的具体病情, 选择合适的中药方剂进行治疗。 2. 针灸疗法: 针灸疗法是中医治疗痛风性肾病的一种方法。通过针灸穴位的刺激, 能够调节身体的平衡, 缓解疼痛和炎症, 同时还可以改善肾功能。 3. 饮食治疗: 饮食治疗是痛风性肾病治疗的重要组成部分。患者应该避免食用高嘌呤食物, 如内脏、肉类、海鲜等, 同时要注意饮食清淡, 避免过度进食。 4. 按摩疗法: 按摩疗法可以缓解痛风性肾病引起的疼痛和炎症。按摩穴位包括足部的太溪穴、脚踝的三阴交穴、膝盖的膝盖穴等。 5. 康复治疗: 康复治疗包括运动疗法、物理疗法等, 可以帮助患者缓解疼痛和炎症, 改善肾功能, 提高生活质量。 需要注意的是, 中医治疗痛风性肾病需要在专业医师的指导下进行, 患者应该积极配合治疗, 按时按量服药, 注意饮食和运动等方面的保健。
HuatuoGPT	痛风性肾病是由于尿酸盐在肾脏中的沉积导致的一种疾病, 中医药对此病的治疗方法主要包括以下几种: 1、泻下疗法: 中医认为, 痛风性肾病的发病与湿热内蕴、瘀血阻滞有关, 因此可以通过泻下疗法来清热利湿、活血化瘀。常用的药物有大黄、芒硝、甘草等。 2、利湿疗法: 中医认为, 痛风性肾病的发病与湿邪有关, 因此可以通过利湿疗法来祛除湿邪。常用的药物有茯苓、泽泻、车前子等。 3、活血疗法: 中医认为, 痛风性肾病的发病与瘀血阻滞有关, 因此可以通过活血疗法来活血化瘀。常用的药物有丹参、红花、桃仁等。 4、补肾疗法: 中医认为, 痛风性肾病的发病与肾虚有关, 因此可以通过补肾疗法来壮肾养血。常用的药物有熟地黄、山茱萸、枸杞子等。 5、降尿酸疗法: 中医认为, 痛风性肾病的发病与尿酸过多有关, 因此可以通过降尿酸疗法来降低血尿酸。常用的药物有别嘌醇、苯溴马隆等。 以上治疗方法需要在医生的指导下进行, 不同的患者可能需要不同的治疗方案。同时, 患者还需要注意饮食控制, 避免食用高嘌呤食物, 如内脏、海鲜、啤酒等, 以帮助控制血尿酸水平, 防止病情进一步恶化。
ChatGLM2-6B	痛风性肾病是一种由高尿酸血症引起的肾脏疾病, 中医药治疗痛风性肾病的方法主要包括以下几种: 1. 清热利湿: 中医药治疗痛风性肾病的重要方法是清热利湿。通过中药治疗, 可以降低体内尿酸水平, 改善肾脏功能。常用的中药有黄柏、金钱草、扁豆、赤芍、川芎等。 2. 祛风通络: 痛风性肾病常伴有风湿痹痛、关节疼痛, 因此中医药治疗痛风性肾病也需祛风通络。常用的中药有独活、秦艽、威灵仙、川芎等。 3. 调节尿酸水平: 中医药治疗痛风性肾病的关键是调节尿酸水平。通过中药治疗, 可以促进尿酸的代谢, 降低尿酸在肾脏的沉积。常用的中药有菊花、玄参、龙骨、牡蛎等。 4. 补肾健脾: 痛风性肾病常伴有肾功能损害, 因此中医药治疗也需补肾健脾。常用的中药有枸杞子、菟丝子、覆盆子、补骨脂等。 5. 活血化瘀: 痛风性肾病可伴有血瘀表现, 中医药治疗可活血化瘀。常用的中药有丹参、川芎、当归、红花等。 中医药治疗痛风性肾病需根据个体情况和病情选择合适的药物和方法。在使用中医药治疗过程中, 需密切观察病情变化, 并定期检查肾功能, 以便及时调整治疗方案。最好在中医药治疗期间, 保持健康的生活习惯, 如合理饮食、适量运动、戒烟限酒等。
ChatGLM2-6B (P-Tuning v2)	痛风性肾病是一种由痛风引起的肾脏疾病, 中医药治疗痛风性肾病的方法主要包括以下几种: 1. 中药治疗: (1) 五味子: 五味子具有清热解毒、消肿止痛、活血化瘀的功效。可以将五味子研成粉末, 每次服用的剂量为1-2克, 每日服用的剂量为3-6克。 (2) 黄连: 黄连具有清热解毒、燥湿泻火、凉血止血的功效。可以将黄连煎汤, 每次服用的剂量为10-15克, 每日服用的剂量为30-60克。 (3) 丹参: 丹参具有活血化瘀、清热解毒、祛风湿的功效, 对痛风性肾病有一定的疗效。可以将丹参煎汤, 每次服用的剂量为10-15克, 每日服用的剂量为30-60克。 (4) 地榆: 地榆具有清热解毒、凉血止血的功效, 对痛风性肾病有一定的疗效。可以将地榆煎汤, 每次服用的剂量为10-15克, 每日服用的剂量为30-60克。 2. 针灸治疗: (1) 关元: 位于腰部以下, 距离肚脐约2-3寸的地方。可以促进血液循环、调节肾功能、缓解疼痛等作用。 (2) 三阴交: 位于小腿内侧, 距离脚踝约1-2寸的地方。可以调节女性生殖系统、缓解疼痛等作用。 (3) 血海: 位于大腿内侧, 距离膝盖约1-2寸的地方。可以活血化瘀、祛风湿、止痛等作用。 3. 饮食调理: 痛风性肾病患者需要避免食用高嘌呤、高脂肪、高盐、高糖的食物, 如肉类、海鲜、酒精等。建议多吃富含蛋白质、纤维、维生素和矿物质的食物, 如鸡肉、鱼类、蔬菜、水果等。
Shengnong- Alpha	中医药在治疗痛风性肾病方面通常采用清热利湿、活血化瘀、通络止痛等治法, 结合辨证论治的原则, 使用多种中药方剂。以下是一些常见的中医药方及其特点: 1.四妙散加减 组成: 苍术、黄柏、牛膝、薏苡仁、丹参、川芎、泽泻等。 功效: 清热利湿、活血通络。 适用症: 湿热型痛风性肾病, 表现为关节红肿热痛、小便短赤、舌苔黄腻等。 2.当归拈痛汤 组成: 当归、羌活、防风、升麻、葛根、白芍、黄芩、黄连、茵陈、甘草等。 功效: 祛风除湿、清热解毒、活血止痛。 适用症: 湿热蕴结、风湿热痹型痛风性肾病, 常伴有腰酸、关节疼痛等症状。 3.桃红四物汤合五苓散 组成: 桃仁、红花、当归、川芎、赤芍、熟地、茯苓、猪苓、泽泻、白术、桂枝等。 功效: 活血化瘀、利水渗湿。 适用症: 血瘀夹湿型痛风性肾病, 常见于慢性期患者, 症状包括关节僵硬、尿少浮肿等。 4.独活寄生汤 组成: 独活、桑寄生、杜仲、牛膝、细辛、秦艽、茯苓、肉桂、防风、川芎、当归、白芍、地黄、人参、甘草等。 功效: 祛风湿、补肝肾、养气血。 适用症: 肝肾不足、风湿痹阻型痛风性肾病, 适用于久病体虚、关节疼痛反复者。 5.大黄䗪虫丸 (现代改良方) 组成: 大黄、䗪虫、水蛭、虻虫、蛴螬、庶虫、桃仁、杏仁、甘草、地黄、白芍、黄芩、柴胡、干漆、䗪虫等。 功效: 活血化瘀、通络止痛。 适用症: 瘀血阻络型痛风性肾病, 适用于高尿酸血症引起的关节肿痛、肾脏功能受损等。 6.萆薢分清饮 组成: 萆薢、石菖蒲、益智仁、乌药、茯苓、泽泻、甘草等。 功效: 利湿通淋、分清化浊。 适用症: 湿热下注型痛风性肾病, 常见于尿频、尿急、尿痛等症状。 7.六味地黄丸合四物汤 组成: 熟地、山茱萸、山药、丹皮、茯苓、泽泻、当归、川芎、白芍、地黄等。 功效: 滋阴补肾、活血化瘀。 适用症: 肾阴亏虚型痛风性肾病, 多见于长期痛风患者, 伴有腰膝酸软、头晕耳鸣等症状。 注意事项: 中医药方的使用应根据患者的具体病情进行辨证施治, 需由中医师指导用药。 痛风性肾病常伴随高尿酸血症和肾功能异常, 中药治疗的同时应配合生活方式调整 (如低嘌呤饮食、控制体重、避免饮酒等) 。 在治疗过程中, 定期监测尿酸水平和肾功能指标非常重要。 如果您需要了解具体的中药配方或进一步的个性化建议, 请咨询专业的中医师或药师。
DeepSeek	痛风性肾病 (尿酸性肾病) 是由于长期高尿酸血症导致尿酸结晶沉积在肾脏, 引发肾小管间质炎症、纤维化, 甚至肾功能衰竭。中医认为其病机主要为 “脾肾亏虚为本, 湿热瘀阻为标”, 治疗需健脾补肾、清热利湿、活血化瘀。以下是具体中医药治疗方案: 一、辨证分型与中药方剂 1. 湿热瘀阻型 (急性期或痛风发作期) 症状: 关节红肿热痛, 小便黄赤、浑浊, 腰酸胀痛, 舌红苔黄腻, 脉滑数。 治法: 清热利湿, 活血通络。 方药: 四妙散合桃红四物汤加减 - 黄柏 10 g (清热燥湿) - 苍术 10 g (健脾燥湿) - 牛膝 15 g (引药下行, 活血通络) - 薏苡仁 30 g (利湿泄浊) - 桃仁 10 g、红花 6 g (活血化瘀) - 土茯苓 30 g、萆薢 15 g (降尿酸、利湿浊) - 车前草 15 g (利尿排酸) 2. 脾肾气虚型 (慢性期或肾功能减退) 症状: 乏力腰酸, 夜尿多, 水肿, 食欲差, 舌淡胖有齿痕, 脉沉弱。 治法: 健脾补肾, 化湿泄浊。 方药: 参苓白术散合济生肾气丸加减 - 黄芪 15 g、党参 15 g (补气健脾) - 白术 10 g、茯苓 15 g (健脾利湿) - 山药 15 g、山茱萸 10 g (补肾固精) - 泽泻 10 g、车前子 10 g (利水消肿) - 丹参 15 g、当归 10 g (活血化瘀) 3. 肾络瘀阻型 (久病肾纤维化) 症状: 面色晦暗, 腰痛固定如针刺, 血肌酐升高, 舌紫暗有瘀斑。 治法: 活血通络, 补肾泄浊。 方药: 大黄䗪虫丸加减 (需医师指导) - 大黄 6 g (泄浊通络, 慎用防伤正) - 䗪虫 6 g、水蛭 3 g (破血逐瘀) - 黄芪 30 g、当归 10 g (补气养血) 二、中成药推荐 1. 痛风定胶囊 (清热利湿, 用于急性期) 2. 尿毒清颗粒 (通腑降浊, 用于肾功能不全) 3. 金水宝胶囊 (补益脾肾, 保护肾功能) 三、外治与辅助疗法 1. 中药灌肠 (用于肾功能不全): - 大黄 10 g、牡蛎 30 g、蒲公英 20 g, 煎水保留灌肠, 帮助排毒。 2. 针灸: - 取穴: 足三里、三阴交、肾俞 (健脾补肾) 、阴陵泉 (利湿) 。 四、食疗与调护 1. 饮食禁忌: - 严格低嘌呤饮食, 忌动物内脏、海鲜、浓汤、酒精。 - 限盐 (每日 ≤ 3 g), 控制蛋白质摄入 (优选鸡蛋、牛奶) 。 2. 推荐药膳: - 薏苡仁粥 (薏苡仁50g + 粳米100g): 健脾利湿。 - 玉米须茶 (玉米须30g煮水): 利尿降尿酸。 五、注意事项 1. 中西结合: 严重肾功能不全 (血肌酐明显升高) 需配合西医治疗 (如降尿酸药、透析) 。 2. 避免伤肾药物: 慎用关木通、广防己等含马兜铃酸的中药。 3. 定期监测: 尿酸、血肌酐、尿蛋白等指标。 建议在肾内科及中医师指导下个体化用药, 尤其活血破瘀药需根据肾功能调整剂量。
XuanHuGPT (LoRA)	痛风性肾病是一种由尿酸盐结晶在肾脏沉积引起的疾病, 属于痛风的一种严重并发症。酸盐结晶会导致肾小球滤过膜的损伤, 从而引起肾功能损害。痛风性肾病常常会导致患者出现血尿、蛋白尿、水肿、高血压等症状, 严重时甚至会导致肾衰竭。根据中医理论, 痛风性肾病的病因主要是由于湿热内蕴、气滞血瘀、风寒湿痹等因素导致。 治疗的方法包括药物治疗、饮食调理、针灸疗法、推拿按摩等。此外, 还可以通过药物熏洗、艾灸疗法和中医膏药疗法等方法来缓解疼痛和改善肾功能。 中药汤剂: 湿热下注型痛风性肾病: 表现为小便短赤、尿急、尿频, 甚则尿血, 腰膝酸软, 或疼痛如灼, 舌红苔黄腻, 脉弦数。治疗方法: 清热利湿, 解毒消肿。方剂: 龙胆泻肝汤。药物: 龙胆草、黄芩、栀子、泽泻、当归、生地黄、柴胡、枳壳。根据湿热的重度和患者体质的差异, 方剂可以调整。例如, 湿热较重者可增加龙胆草和黄芩的剂量, 湿热轻微者可适当减少。 风寒湿阻型痛风性肾病: 表现为腰膝酸冷疼痛, 局部肿胀, 小便不利, 或尿中有沉淀, 舌苔白腻, 脉沉。治疗方法: 祛风除湿, 活血通络。方剂: 四逆汤。药物: 人参、甘草、干姜和附子。 气滞血瘀型痛风性肾病: 表现为腰膝酸软疼痛, 疼痛难以忍受, 局部有明显肿胀, 小便不利, 或尿中有血, 舌质紫暗, 脉弦。治疗方法: 活血化瘀, 通络止痛。方剂: 血府逐瘀汤加减。药物: 桃仁、红花、白芍、川芎、丹参、赤芍、柴胡、枳壳、牛膝。 阴阳两虚型痛风性肾病: 表现为腰膝酸软疼痛, 疼痛难以忍受, 小便不利, 或尿中有沉淀, 腰膝酸软, 或腰膝冷痛, 舌质淡, 脉沉。治疗方法: 补肾益阴, 活血通络。方剂: 左归丸合肾气丸加减。药物: 熟地黄、山药、枸杞子、菟丝子、天冬、山茱萸、白芍、川芎、白术、茯苓。若患者表现为虚寒较重, 则可加用附子、肉桂等温阳药物, 改善寒湿症状。 2. 饮食调理: 痛风性肾病患者应避免高嘌呤饮食, 如肉类、海鲜、啤酒等。多吃富含维生素、矿物质和膳食纤维的食物, 如蔬菜、水果、全谷类等。 3. 针灸疗法: 针灸疗法通过刺激人体的穴位, 调整气血循环, 降低尿酸水平, 缓解疼痛和改善肾功能, 对痛风性肾病有一定的疗效。常用的穴位有血海、三阴交、足三里等。 4. 推拿按摩: 推拿按摩作为一种非药物治疗方法, 在中医药治疗痛风性肾病的过程中也可以发挥重要作用。在治疗痛风性肾病时, 可以选择按摩腰部、腿部等部位, 以达到缓解疼痛、改善血液循环等作用。 需要注意的是, 中医药治疗痛风性肾病应在专业医生的指导下进行, 患者应按照医生的建议进行治疗, 并定期复查肾功能。同时, 患者在治疗过程中应保持良好的生活习惯, 如保持规律作息, 避免过度劳累、劳累等。
Reference Answer: TCM Experts[43]	中药汤剂治疗痛风性肾病主要包括以下几种方法: 1.清热解毒法适用于痛风性肾病属于湿热内蕴证的患者。以关节肿胀, 尿黄, 便秘, 苔黄腻等为主要症状。治疗时常用黄柏 (主肾经, 清热燥湿, 泻火解毒、保肝利胆) 、黄连 (归脾胃、肝、胆、心、大肠经, 清热, 解毒, 燥湿泻火) 、栀子 (归心、肺、三焦经, 清热利湿, 泻火除烦, 凉血解毒) 等药物, 具有清热解毒、燥湿止泻的功效。 2.活血化瘀法适用于痛风性肾病属于血瘀证的患者。以关节疼痛, 肌肤甲错, 舌质紫暗等为主要症状。治疗时常用丹参 (归心、肝经, 祛瘀止痛, 养血安神, 凉血消痈) 、川芎 (归肝、胆、心包经, 祛风止痛、活血化瘀、活血行气) 、桃仁 (归心、肝、大肠经, 活血化瘀, 止咳平喘、润肠通便) 等药物, 具有活血化瘀、通络止痛的功效。 3.强筋骨法适用于痛风性肾病属于肝肾亏损证的患者。以关节酸软, 腰膝疼痛, 头晕耳鸣等为主要症状。治疗时常用杜仲 (归肝、肾经, 补肝肾, 促消化, 降血压) 、牛膝 (归肝、肾经, 活血祛瘀, 补肝肾, 强筋骨, 利尿通淋) 、熟地黄 (归肾、心经, 补血滋润、益精填髓) 等药物, 具有补肝肾、强筋骨的功效。 4.调节阴阳法适用于痛风性肾病属于阴阳失衡证的患者。以关节肿痛, 腰膝酸软, 畏寒发热等为主要症状。治疗时常用附子 (归心、肾、脾经, 上助心阳, 中温脾阳, 下补肾阳) 、肉桂 (主肾、脾、膀胱经, 补元阳、温脾胃、除积冷、通血脉) 、熟地黄 (归肾、心经, 补血滋润、益精填髓) 等药物, 具有调节阴阳、平衡气血的功效。 在治疗痛风性肾病时, 选择毫针疗法和电针疗法治疗。主要在选择关元穴、足三里穴、太冲穴、肾俞穴、命门穴等处进行针灸治疗。在中医药治疗痛风性肾病的过程中, 推拿按摩作为一种非药物治疗方法。

### Inquiry on prescription evaluation

For children with asthma in the remission stage, during treatment using a traditional Chinese medicine prescription aimed at tonifying Qi, activating blood circulation, and calming asthma, is it effective to add Poria (Fu Ling) and Tangerine Peel (Chen Pi) if the child presents with Kidney Qi deficiency?

(对于小儿哮喘缓解期患儿, 在进行治疗的过程中采用中医益气活血定喘方进行治疗, 如果患儿表现为肾气虚弱, 为患儿加茯苓、陈皮是否有效?).

**Table 9:** Inquiry on prescription evaluation

LLMs	Response
MING-MOE	益气活血定喘方是一种中医治疗哮喘的方法, 其中包含中药成分茯苓、陈皮等。对于小儿哮喘缓解期患儿, 在进行治疗的过程中采用中医益气活血定喘方进行治疗, 如果患儿表现为肾气虚弱, 加茯苓、陈皮可能具有一定的治疗效果。 茯苓、陈皮等中药具有健脾、化痰、理气的功效, 可以帮助缓解哮喘症状, 改善患儿的消化系统和呼吸道系统功能。此外, 茯苓、陈皮等中药还具有一定的降气作用, 可以帮助缓解哮喘引起的呼吸困难等症状。 但是, 加茯苓、陈皮是否有效需要根据具体情况而定。如果患儿的肾气虚弱是由于其他疾病所引起, 例如慢性肾脏疾病等, 加茯苓、陈皮可能并不能够有效治疗哮喘。在这种情况下, 应该先治疗其他疾病, 然后再考虑加入茯苓、陈皮等中药进行治疗。 对于小儿哮喘缓解期患儿, 在进行治疗的过程中应该根据个体差异制定个性化的治疗方案, 包括中药成分的选择和使用。如果患儿表现为肾气虚弱, 应该先治疗其他疾病, 然后再考虑加入茯苓、陈皮等中药进行治疗。
HuatuoGPT	小儿哮喘是一种常见的儿童呼吸系统疾病, 中医治疗小儿哮喘有其独特的优势。益气活血定喘方是中医中常用的治疗小儿哮喘的方剂, 具有益气补肾、活血化痰、定喘止咳的作用。 对于肾气虚弱的小儿哮喘患儿, 加茯苓、陈皮确实是一种有效的治疗方法。茯苓具有利水渗湿、健脾和胃、安神止惊的作用, 适合用于治疗肾气虚弱导致的小儿哮喘。陈皮则具有理气健脾、燥湿化痰的作用, 可以改善肾气虚弱导致的痰多、咳嗽等症状。 然而, 每个患儿的体质和病情都有所不同, 治疗方案需要根据患儿的具体情况进行个体化调整。建议在使用中药治疗时, 应咨询专业中医医生, 根据患儿的病情、体质、年龄等因素进行综合评估, 制定出适合的治疗方案。同时, 患儿在治疗过程中应定期进行复查, 以监测病情的变化和治疗效果。
ChatGLM2-6B	小儿哮喘缓解期的治疗通常涉及缓解症状和改善肺功能。中医在治疗小儿哮喘方面有着悠久的历史, 其中益气活血定喘方是一种常用的方剂, 主要用于缓解哮喘症状, 改善患儿的肺功能和体质。 如果患儿表现为肾气虚弱, 根据中医理论, 这可能影响哮喘的治疗效果。在这种情况下, 加入茯苓和陈皮可能是有益的。茯苓具有利水渗湿、健脾安神的作用, 有助于增强患儿的体质和改善肾气虚弱症状。陈皮则能理气健脾、燥湿化痰, 有助于改善患儿的肺功能和缓解哮喘症状。 然而, 需要强调的是, 中医治疗哮喘的方法应根据患儿的个体情况来调整。在使用任何中药方剂之前, 建议咨询专业的中医师, 以确保安全和有效性。同时, 中医治疗应与西医治疗相结合, 以提供更全面和有效的治疗方案。
ChatGLM2-6B (P-Tuning v2)	从中医理论来看, 小儿哮喘缓解期的治疗通常需要考虑患儿的体质和具体症状。中医认为哮喘与肺、脾、肾三脏的功能失调有关, 尤其强调肾为先天之本, 对哮喘患儿的治疗和预防有重要作用。 益气活血定喘方是一种中医方剂, 主要用于治疗哮喘, 通过益气、活血、定喘来调整患儿的身体状态。如果患儿表现为肾气虚弱, 可能会出现气短、乏力、面色苍白、小便清长等症状。在这种情况下, 加入茯苓和陈皮可能是有益的。 茯苓具有利水渗湿、健脾安神的作用, 有助于改善患儿的脾肾功能, 增强体质。陈皮则能理气健脾、燥湿化痰, 对于哮喘患儿的脾虚湿盛、痰湿内阻等症状有一定的改善作用。 然而, 需要注意的是, 中医治疗强调个体差异和辨证施治。每个患儿的体质和病情都有所不同, 因此在实际应用中, 是否加入茯苓和陈皮以及剂量调整, 应该由具备中医专业知识的医师根据患儿的实际情况来决定。此外, 中医治疗通常需要结合患儿的饮食、生活习惯等多种因素进行综合调理, 以达到最佳的治疗效果。
XuanHuGPT (LoRA)	小儿哮喘缓解期患儿在中医治疗中, 采用益气活血定喘方进行治疗。益气活血定喘方是一种具有益气养血、活血化瘀、定喘止咳作用的方剂, 常用于治疗小儿哮喘、慢性咳嗽等病症。然而, 如果患儿在治疗过程中表现为肾气虚弱, 那么加茯苓、陈皮是否有效呢?我们来进行分析。 1. 茯苓: 茯苓具有利水渗湿、健脾和胃、宁神安心的功效。在中医理论中, 茯苓入脾、心经, 具有健脾利湿、化痰祛湿的作用, 同时也有一定的安神作用。对于肾气虚弱的小儿哮喘缓解期患者, 茯苓的加入可以帮助增强脾胃功能, 但不能直接补肾。 2. 陈皮: 陈皮具有理气健脾、燥湿化痰的功效。在中医理论中, 陈皮入脾、肺经, 具有健脾和胃、燥湿化痰的作用, 常用于治疗脘腹胀满、恶心、食欲不振等问题。对于肾气虚弱的小儿哮喘缓解期患者, 陈皮的加入可以帮助增强脾胃功能, 促进水湿的代谢, 但不能直接补充肾气。 3. 肾气虚弱: 肾气虚弱是指肾脏功能减退, 表现为腰膝酸软、头晕目眩、失眠多梦等症状。在中医理论中, 肾气与生长发育、骨骼健康等密切相关。对于小儿哮喘缓解期患者, 肾气虚弱可能会影响病情的恢复。 4. 方剂配合: 在治疗小儿哮喘缓解期时, 采用益气活血定喘方进行治疗。在治疗过程中, 茯苓和陈皮可以作为辅助药物, 改善脾胃功能、理气化痰, 但它们并不直接补充肾气。需要加入其他中药材来增强肾气的功能。例如, 可以加入补肾益气的中药材, 如枸杞子、女贞子、肉桂等, 以及固表益气的药材, 如黄芪、党参等。这些中药材可以协同作用, 更好地治疗肾气虚弱的小儿哮喘缓解期患者。 需要注意的是, 药物的使用需要根据患儿的具体情况和医生建议来进行。在治疗过程中, 需要密切观察患儿的症状变化, 并根据需要进行相应的调整。同时, 患儿在治疗期间也需要注意饮食调理、生活作息和锻炼身体等方面, 以促进病情的恢复。
Shennong- Alpha	一、肾气虚弱的表现: 腰膝酸软: 小儿常感到腰部无力、腿脚沉重。 畏寒肢冷: 手脚冰凉, 怕冷。 尿频或夜尿多: 夜间频繁排尿, 白天也容易尿急。 发育迟缓: 身高、体重增长缓慢。 精神不振: 易疲劳, 注意力不集中。 舌淡苔白: 舌色偏淡, 舌苔薄白。 脉沉细弱: 脉搏较弱, 提示肾气不足。 二、茯苓与陈皮的作用: 1. 茯苓 ([[nmm01eg]]) 功效: 健脾利湿, 宁心安神。 在肾气虚弱中的作用: 肾气虚弱常伴随脾虚, 茯苓可健脾化湿, 改善脾肾两虚的状态。 有助于调节水液代谢, 减少因肾气不足导致的水肿或尿频。 2. 陈皮 ([[nmm00jg]]) 功效: 理气健脾, 燥湿化痰。 在肾气虚弱中的作用: 痰湿内生是哮喘反复的重要原因, 陈皮能行气化痰, 减轻痰湿对肺的影响。 同时, 陈皮有助于调和脾胃, 增强药物吸收, 提升整体疗效。 三、为什么加茯苓和陈皮有效? 1. 健脾补肾: 肾气虚弱常伴脾虚, 茯苓健脾以助肾, 达到 “土旺而金生”的效果。 2. 化痰止咳: 陈皮理气化痰, 有助于缓解哮喘缓解期的痰湿阻肺症状。 3. 调和药性: 茯苓和陈皮可以调和益气活血类药物 (如黄芪、党参等), 避免过于温燥或滋腻。 四、建议配伍与注意事项: 推荐配伍: 益气活血定喘方基础药物 (如黄芪、党参、丹参、川芎等) + 茯苓、陈皮 (可适量加入) 注意事项: 若患儿肾阳虚明显 (如怕冷、四肢冰凉、舌淡苔白), 可考虑加入附子、肉桂等温肾助阳之品。 若有阴虚火旺表现 (如口干、烦躁、舌红少苔), 则不宜过用温燥药物, 应以滋阴清热为主。 儿童用药需注意剂量, 建议在专业中医师指导下使用。 五、总结: 在小儿哮喘缓解期, 若患儿表现为肾气虚弱, 在益气活血定喘方中加入茯苓和陈皮是合理且有效的, 因为它们能够健脾利湿、理气化痰, 辅助改善肾气虚弱及痰湿内阻的情况。但具体用药仍需根据患儿的整体体质和症状进行个体化调整。
DeepSeek	在中医理论中, 小儿哮喘缓解期若表现为肾气虚弱, 治疗应以补肾纳气、温阳固本为主。若此时单纯加用茯苓、陈皮 (健脾化痰、理气燥湿之品), 可能效果有限, 甚至需谨慎配伍。以下是具体分析: 1. 茯苓、陈皮的药性与适用性 茯苓: 性味甘淡平, 归心、肺、脾、肾经, 主要功效为利水渗湿、健脾宁心。 适用于脾虚湿盛或肺脾两虚导致的痰湿内停, 但无直接补肾作用。 陈皮: 性味辛苦温, 归脾、肺经, 主要功效为理气健脾、燥湿化痰。 适用于气滞痰阻或脾胃湿困, 但性偏温燥, 可能耗气伤阴, 肾虚患儿需注意。 结论: 两药均偏重调理脾肺, 对肾气虚弱的核心病机 (肾不纳气、阳气不足) 针对性不足。 2. 肾气虚弱型哮喘的辨证要点 典型表现: 喘息气短、动则加重, 腰膝酸软, 畏寒肢冷, 夜尿频多, 舌淡苔白, 脉沉细。 核心病机: 肾阳不足, 摄纳无权, 气不归元。 治疗原则: 主方: 金匮肾气丸、右归丸等加减 (如加蛤蚧、补骨脂、淫羊藿等温肾纳气) 。 慎用: 过度理气化痰药 (如陈皮) 可能耗散肾气, 利湿药 (如茯苓) 需配伍温阳药。 3. 茯苓、陈皮的合理配伍建议 若患儿兼有脾虚湿盛或痰湿内阻 (如舌苔白腻、痰多稀白), 可在补肾基础上少量配伍: 示例方: 益气活血定喘方 + 补骨脂、淫羊藿 (温肾) + 茯苓 (健脾渗湿) + 陈皮 (少量, 佐制滋腻) 。 注意事项: 陈皮用量宜轻 (36 g), 避免辛燥伤阴。 茯苓可配伍山药、白术, 增强健脾作用而不伤肾气。 4. 更推荐的中药加减 针对肾气虚弱, 建议优先选择以下药物: 温肾纳气: 蛤蚧、紫河车、核桃仁、补骨脂。 益气固表: 黄芪、太子参 (防外感诱发哮喘) 。 活血通络: 丹参、当归 (兼顾 “益气活血”原方思路) 。 总结 单纯加茯苓、陈皮: 效果有限, 可能偏离肾虚主证。 合理配伍: 在温肾基础上佐以茯苓、陈皮 (需辨证是否有痰湿兼证) 。 关键: 需结合患儿具体症状, 由中医师调整方药, 避免 “见痰治痰”而忽视根本。 建议临床中四诊合参, 尤其关注舌脉 (如肾虚者多见舌淡胖、脉沉弱), 灵活加减。
Reference Answer: TCM Experts[44]	患儿选择采用益气活血定喘方进行治疗, 该药物的具体配方为: 川芎、地龙、紫菀、桃仁、当归尾、僵蚕、苦杏仁各6 g, 黄芪20 g, 毛青豆15 g。 对患儿治疗时随症加减, 如果患儿表现为脾气虚弱, 为患儿加茯苓15 g, 陈皮6 g; 如果患儿表现为肺气虚弱, 需为患儿加太子参10g, 五味子6g; 如果患儿表现为肾气虚弱, 为患儿加补骨脂10 g, 女贞子10 g。 药物用水煎服取汁, 每日为患儿给药1剂, 分两次温服。连续为患儿每周服药5日, 治疗三个月作为一个疗程, 如果患儿治疗过程中存在哮喘发作, 可为患儿进行对症处理。

## Abbreviations

BLEU: Bilingual Evaluation Understudy
LCS: Longest Common Subsequence
LLMs: Large Language Models
LoRA: Low-Rank Adaptation
MLA: Multi-Head Latent Attention
MOE: Mixture of Experts
MoLoRA: Mixture of Low-Rank Adaptation
NMPA: National Medical Products Administration
PEFT: Parameter-Efficient Fine-Tuning
RAG: Retrieval-Augmented Generation
RLHF: Reinforcement Learning with Human Feedback
ROUGE: Recall-Oriented Understudy for Gisting Evaluation
TCM: Traditional Chinese Medicine
